# Phosphorylation of the Hepatitis B Virus Large Envelope Protein

**DOI:** 10.3389/fmolb.2021.821755

**Published:** 2022-02-23

**Authors:** Marie-Laure Fogeron, Lauriane Lecoq, Laura Cole, Roland Montserret, Guillaume David, Adeline Page, Frédéric Delolme, Michael Nassal, Anja Böckmann

**Affiliations:** ^1^ Molecular Microbiology and Structural Biochemistry, Labex Ecofect, UMR 5086 CNRS/Université de Lyon, Lyon, France; ^2^ Protein Science Facility, SFR BioSciences CNRS UAR 3444, Inserm US8, UCBL, ENS de Lyon, Lyon, France; ^3^ Department of Medicine II / Molecular Biology, Medical Center, University Hospital Freiburg, University of Freiburg, Freiburg im Breisgau, Germany

**Keywords:** hepatitis B, L HBsAg, phosphorylation, NMR, cell-free (CF) protein synthesis, mass spectrometry

## Abstract

We here establish the phosphorylation sites in the human hepatitis B virus (HBV) large envelope protein (L). L is involved in several functionally important interactions in the viral life cycle, including with the HBV cellular receptor, HBV capsid, Hsc70 chaperone, and cellular membranes during fusion. We have recently shown that cell-free synthesis of the homologous L protein of duck HBV in wheat germ extract results in very similar phosphorylation events to those previously observed in animal cells. Here, we used mass spectrometry and NMR to establish the phosphorylation patterns of human HBV L protein produced by both *in vitro* cell-free synthesis and in *E. coli* with the co-expression of the human MAPK14 kinase. While in the avian virus the phosphorylation of L has been shown to be dispensable for infectivity, the identified locations in the human virus protein, both in the PreS1 and PreS2 domains, raise the intriguing possibility that they might play a functional role, since they are found at strategic sites predicted to be involved in L interactions. This would warrant the further investigation of a possible function in virion formation or cell entry.

## Introduction

The three hepatitis B virus (HBV) envelope proteins L, M, and S [large, middle and small hepatitis B surface antigen (HBsAg)] form the viral envelope. S is an integral membrane protein for which structural models predict four transmembrane-spanning helices, interrupted by an N-proximal cytosolic loop and, after the second helix, the antigenic loop, which presents a complex yet poorly defined structure stabilized by multiple disulfide bridges. M and L share their C-termini with S but carry in addition the PreS1 plus PreS2 (for L) and PreS2 (for M) domains. PreS1 and PreS2 together, collectively termed PreS, are suspected to represent an intrinsically disordered protein domain. This was experimentally supported for the avian (duck) homolog, DHBV PreS (Urban et al., 2000), while the corresponding data for PreS from human HBV lacked.

The PreS part of L plays a central role in a variety of interactions with viral and cellular proteins. The very N-terminal residues of PreS represent a first important site, with the first 48 amino acids involved in binding to the HBV receptor (Glebe et al., 2005; Gripon et al., 2005), the sodium taurocholate co-transporting polypeptide (NTCP) (Yan et al., 2012). N-terminal myristoylation is central in this context (Persing et al., 1987). Further down the PreS sequence, several hydrophobic stretches can be identified that are concentrated between residues 50–70. Most models favor that different residues distributed along preS are responsible for membrane interaction, hinting at a possible fusion mechanism (Núñez et al., 2009; Delgado et al., 2015), but a recent proposal locates the fusion peptide exactly to this hydrophobic region (Pérez-Vargas et al., 2021). A third region of interest is localized at the PreS1/PreS2 border, comprising residues between approximately amino acids 90–120, which is believed to be involved in interactions with the viral capsid during particle formation (Gudima et al., 2007; Xi et al., 2021). Finally, another site in PreS1 is involved in the interaction with the Hsc70 chaperone, reported to be a determinant in the i-PreS orientation observed in immature viral particles (Prange et al., 1999; Prange, 2012). In sum, several specific sites in preS have been identified to be involved in the important functional interactions of L.

Post-translational modifications have been shown to play a central role in the HBV life cycle (Heger-Stevic et al., 2018; Yang, 2018). The phosphorylation of PreS has been thoroughly investigated in the DHBV L variant (Grgacic and Anderson, 1994; Grgacic et al., 1998). Metabolic labeling with ^32^P and digestion with residue-specific phosphatases (Grgacic and Anderson, 1994) have identified several phosphorylation sites, whose functional importance has, in part, been assessed by the mutational analysis of Ser and Thr residues (Grgacic et al., 1998). Mutations mimicking absent or constitutive PreS phosphorylation had no detectable effect on DHBV replication (Grgacic et al., 1998), although DHBV L phosphorylation was found important in host cell–virus cross talk (Rothmann et al., 1998).

We have recently found, during the wheat-germ cell-free protein synthesis of DHBs proteins for structural studies, that the DHBV L protein is phosphorylated in this system (David et al., 2018; David et al., 2019). While it was known that the cell-free extract indeed contains kinases, allowing post-translational phosphorylation (Badillo et al., 2017), our analysis of the DHBV L protein confirmed that the phosphorylation sites are the same as in animal cells (Grgacic et al., 1998) and identified two additional sites (David et al., 2019).

As an important step in the ongoing studies in our group on HBV preS–capsid interactions, we here investigate preS phosphorylation, in the protein, from the human virus, as obtained in recombinant systems, and apply a similar analysis to the HBV L protein and PreS fragments thereof. Based on the results, we designed a bacterial kinase co-expression system able to phosphorylate PreS, which we show to reproduce the results obtained in the acellular system. In addition, we used NMR to structurally analyze the PreS protein and to confirm major phosphorylation sites by NMR chemical-shifts. Our approach confirmed the disordered nature of the protein, and identified four consensus sites as well as several additional potential PreS phosphorylation sites.

## Materials and Methods

### Plasmids

For cell-free expression, PreS1, PreS, and full-length HBV L sequences were amplified by PCR from the HBV isolate H2815 (genotype D5, GenBank Accession Number KP322603.1), and cloned into a pEU-E01-MCS vector (CellFree Sciences, Matsuyama, Japan). A *Strep*-tag II, shortly named “tag” in the following, was fused either to the N- or C-terminal end for purification (Schmidt and Skerra, 2007), resulting in the four constructs PreS1_tag_, _tag_PreS, PreS_tag_, and _tag_L. The plasmids were amplified in *Escherichia coli* TOP10 cells (Life Technologies, Carlsbad, CA, USA). DNA was isolated using a NucleoBond Xtra Maxi kit (Macherey-Nagel). Plasmids were further purified by phenol/chloroform extraction, according to CellFree Sciences recommendations.

For the bacterial expression of PreS (^*E.coli*^PreS_tag_), cDNA-encoding PreS was cloned into a pRSF-T7 vector and into an analogous pRSF-T7 vector carrying a MAPK14 gene under a Tet promotor in order to promote PreS phosphorylation. A solubility-enhancing fusion protein, GB1, with an N-terminal His-Tag, was fused at the N-terminus of PreS and a *Strep*-tag II to its C-terminus. A TEV protease cleavage site was inserted between GB1 and PreS, resulting in the following constructs: pRSF_T7-H6-GB1-ENLYFQG-preS-*Strep*-tag-II and pRSF_Tet-H6MAPK14_T7-H6-GB1-ENLYFQG-preS-*Strep-*tag-II. Plasmids were amplified in *E. coli* TOP10 cells (Life Technologies). DNA was isolated using QIAprep Spin Miniprep Kit (Qiagen, Hilden, Germany).

### Wheat Germ Cell-free Expression and Purification of PreS and L

Homemade wheat germ extract was prepared using non-treated durum wheat seeds (Semences du Sud, Vic-Fezensac, France), as described in Fogeron et al. (2017). Protein synthesis was performed with uncoupled transcription and translation. Transcription was performed using 100 μg/ml plasmid, 2.5 mM NTP mix (Promega, Charbonnières-les-Bains, France), 1 U/μl RNase inhibitor (CellFree Sciences, Matsuyama, Japan), and 1 U/μl SP6 RNA polymerase (CellFree Sciences, Matsuyama, Japan) in transcription buffer (CellFree Sciences, Matsuyama, Japan) composed of 80 mM HEPES-KOH pH 7.6, 16 mM magnesium acetate, 10 mM DTT, and 2 mM spermidine in nuclease-free water. The solution was incubated for 6 h at 37°C; the produced mRNA solution was then used directly for translation. Translation was performed using the bilayer method (Takai et al., 2010; Fogeron et al., 2015), either at a small scale with the one well of a 6-well plate (6 ml total reaction volume), or at a large scale with two 6-well plates (2 × 36 ml total reaction volume) in order to obtain a sufficient amount of protein to perform NMR experiments. The feeding buffer composition was 30 mM HEPES-KOH pH 7.6, 100 mM potassium acetate, 2.7 mM magnesium acetate, 16 mM creatine phosphate, 0.4 mM spermidine, 1.2 mM ATP, 0.25 mM GTP, and 4 mM DTT supplemented with 6 mM amino acid mix (0.3 mM average concentration per amino acid). The translation mix was prepared with the mRNA solution, wheat germ extract (250 μl for each well of a 6-well plate), 6 mM amino acid mix, and 40 μg/ml creatine kinase. On the bottom of the each well, the translation mix (518 μl for each well) was then deposited under the feeding buffer (5.5 ml for each well), allowing for the formation of a bilayer. The plate was incubated overnight at 22°C without shaking. For NMR sample preparation, large-scale production was performed in presence of a mixture of (^15^N) or (^2^H-^13^C-^15^N)-labeled amino acids (Cambridge Isotope Laboratories) added to the reaction solution and the feeding buffer. A summary of the different sample preparations is given in Supplementary Table S1.

For *Strep*-Tactin affinity chromatography, the total cell-free reaction was incubated with homemade benzonase 250 U/μl (50 μl per well) on a rolling wheel for 30 min at room temperature. This solution was then centrifuged at 20,000 *g*, 4°C for 30 min. The supernatant obtained was loaded either on a 200-μl (small-scale production) or on two 1-ml (large-scale production) *Strep*-Tactin Superflow® gravity flow columns (IBA Lifesciences, Göttingen, Germany). Purification was performed as described previously (Fogeron et al., 2015; Fogeron et al., 2016). The protein of interest was eluted in 100 mM Tris-HCl pH 8.0, 150 mM NaCl, 1 mM EDTA, and 2.5 mM D-desthiobiotin (IBA Lifesciences, Göttingen, Germany).

All experiments were assessed using 15% polyacrylamide SDS-PAGE gels. Samples were resuspended in a loading buffer containing 62.5 mM Tris-HCl pH 6.8, 10% glycerol (v/v), 2% SDS (w/v), 5% β-mercaptoethanol (v/v), and 0.01% bromophenol blue (w/v).

### Bacterial Expression and Purification of ^E.coli^PreS_tag_

*E. coli* BL21 (DE3) or *E. coli* BL21*CP (DE3) cells were transformed with the plasmids for PreS expression or PreS plus MAPK14 co-expression, respectively, and grown at 37°C, either in LB (Lysogeny Broth) for purification setup and mass spectrometry or in M9 minimal medium containing 2 g/L of ^13^C-labeled glucose and ^15^N-labeled ammonium chloride for NMR spectroscopy. T7 promoter-controlled protein expression was induced at an OD_600nm_ of 1.2 using 1 mM of isopropyl-ß-D-1-thiogalactopyranoside (IPTG) for 17 h at 30°C and cells were harvested (6,000 *g*, 20 min, 4°C). Purification procedures were the same for labeled and unlabeled samples. Cells were resuspended in 4 ml/g of cell pellet lysis buffer (20 mM NaPO_4_, pH 7.5, 500 mM NaCl, 40 mM imidazole) supplemented with EDTA-free protease inhibitor (Roche). Cell lysis was performed by incubating with 1 mg/ml of lysozyme (Sigma) for 50 min at 4°C under rotation, and nucleic acids were digested with Benzonase nuclease 250 U/µl (6 μl/L of culture) supplemented with 2 mM MgCl_2_, for 30 min at room temperature. Cellular membranes were broken by passing three times through a microfluidizer (Microfluidics M-110P) at 15,000 psi. Soluble proteins were isolated by centrifugation (25,000 *g*, 30 min, 4°C) and filtrated using a 0.45 µm filter, before being loaded into a 5 ml HisTrap (GE Healthcare) affinity column, connected to a Biorad NGC chromatography system. The HisTrap column was washed with lysis buffer until A_280nm_ returned to baseline, and His-tagged proteins were eluted through a one-step elution with elution buffer (20 mM NaPO_4_ pH 7.5, 500 mM NaCl, 1 M imidazole). The eluted proteins were dialyzed using a 3.5 kDa cut-off membrane (Spectrum labs) in TEV protease reaction buffer (50 mM Tris-HCl pH 7.5, 1 mM EDTA, 5 mM DTT), at 4°C under slow stirring. GB1 fusion proteins were cleaved using homemade TEV protease 10 U/µl (0.2 ml or 2,000 U per milligram of PreS protein) by incubating overnight at 4°C under rotation. Cleaved PreS protein was recovered using *Strep*-Tactin resin (IBA Lifesciences), in batch mode, following the manufacturer’s recommendations. EDTA-free protease inhibitor (Roche) 1X was added to the eluted protein.

PreS was dialyzed using a 3.5 kDa cut-off membrane (spectrum labs) overnight in final NMR buffer (20 mM NaPO_4_ pH 6, 50 mM NaCl). Protein concentration was determined using a Nanodrop instrument (Thermo Fisher) and the absorbance at 280 nm. Subsequently, the protein solution was concentrated by immerging the dialysis bag containing the protein and protease inhibitor into Sephadex G-25 powder at 4°C. Concentration was followed by weighing the dialysis bag before adding the powder and every 12–20 h thereafter. This approach was used to avoid PreS sticking to cellulose membranes used in concentrators as Amicon (Merck) or Vivaspin (Sartorius). The protein concentration was measured by Nanodrop before storage at −80°C. Protein solutions in all purification steps were analyzed using 15% polyacrylamide SDS-PAGE gels. The typical yields of PreS and PreS-MAPK14 co-expressed protein were around 30 mg in LB and 10 mg per liter of M9 medium culture.

### Solution-State NMR Spectroscopy

Isotopically labeled samples were dialyzed in 20 mM HEPES-KOH pH 7.5 containing 50 mM NaCl for ^15^N- and ^2^H-^13^C-^15^N-PreS1_tag_, in 20–50 mM phosphate buffer pH 6.0 containing 50 mM NaCl for ^2^H-^13^C-^15^N-PreS_tag_ and _tag_PreS produced in cell-free, and ^13^C-^15^N-^*E.coli*^PreS_tag_ proteins ± MAPK14 produced in the bacterial expression system. A pH of 6.0 could not be used for PreS1_tag_ due to its close theoretical isoelectric point of 6.37, which could result in protein aggregation. D_2_O was added to a final volume of 7%, and protein concentration was quantified by NanoDrop. Concentrations were estimated for cell-free samples at 150 µM for PreS1_tag_, 60 µM for PreS_tag_ and 120 µM for _tag_PreS, and 50 µM for both bacterial samples MAPK14-^*E.coli*^PreS_tag_ and ^*E. coli*^PreS_tag_. About 1 µl of 2,2-dimethyl-2-silapentane-5-sulfonate (DSS) was added to each sample for chemical-shift referencing. NMR experiments were recorded at 298 K (25°C) on Bruker Avance II spectrometers operating at 600 MHz (_tag_PreS and ^*E. coli*^PreS_tag_ samples) and 950 MHz (PreS1_tag_ and PreS_tag_). Backbone resonances were assigned using two-dimensional (2D) BEST-TROSY spectra and three-dimensional (3D) BEST-TROSY versions of HNCA, HNcaCO, as well as HNCACB_2H, HNcoCACB_2H, and HncaCO_2H optimized for deuterated proteins (cell-free samples) (Solyom et al., 2013) and HNCACB, HNcoCACB, and HncaCO for non-deuterated proteins (^*E.coli*^PreS_tag_ samples). Pulse sequences were installed on the 600 MHz using the NMRlib tool (Favier and Brutscher, 2019). For details on NMR samples, NMR experiments, and assignment, see Supplementary Table S2. All PreS samples started to show degradation products in the NMR spectra after 1 week, even in the presence of a protease inhibitor. NMR data were processed using TopSpin 4.0.7 (Bruker) and analyzed with CcpNmr Analysis 2.4.2 (Vranken et al., 2005; Stevens et al., 2011).

For the secondary chemical shifts, the Cα and Cβ chemical shifts of each residue for PreS1 and both PreS constructs were compared to their random coil shift taken from Wang and Jardetzky (2002). The difference between ΔCα and ΔCβ were calculated, where positive values indicate the tendency to form an α-helix and negative values indicate the tendency to form a β-strand.

### MALDI-TOF Mass Spectrometry

Mass spectra were acquired with a Voyager-DE PRO (Sciex, Framingham, MA, USA) equipped with a nitrogen laser emitting at 337 nm. Ions were accelerated to a final potential of 20 kV, and the mass spectrum was the sum of 300 laser shots. An external mass calibration was used (a mixture of peptides from the Sequazyme™ standards kit, AB Sciex). The analysis was performed in linear mode (instrumental mass accuracy is 0.05%). Samples were prepared by diluting 10-fold the protein solution (0.2 mg/ml) in the matrix sinapinic acid (Sigma-Aldrich, St. Louis, MI, USA), used without further purification and dissolved in 0.1%TFA/acetonitrile (70/30 v/v). About 1 μl of the mixture was deposited onto the MALDI sample plate and let dried to complete co-crystallization.

### Nano LC-MS/MS Analysis

The solutions of all purified PreS1 and PreS samples were digested overnight at 37°C with 1/100 the amount (w/w) of trypsin (trypsin porcine; Promega, Charbonnières-les-Bains, France). _tag_L protein solution was reduced with 5 mM TCEP for 45 min at 57°C, then alkylated with 10 mM iodoacetamide for 30 min in the dark at room temperature and under agitation (850 rpm) and digested overnight at 25°C with chymotrypsin 1/100 ratio (bovine pancreas chymotrypsin; Promega, Charbonnières-les-Bains, France). Peptide digests were desalted using C18 spin columns (Thermo Scientific). Peptides were dried in a speed-vac and suspended in 0.1% HCOOH. Samples were analyzed using an Ultimate 3,000 nano-RSLC (Thermo Scientific) coupled on line with a Q-Exactive HF mass spectrometer *via* a nano-electrospray ionization source in positive ionization mode (Thermo Scientific). Peptide mixtures were loaded on a C18 Acclaim PepMap100 trap-column and then separated on a C18 Acclaim Pepmap100 nano-column 50 cm × 75 μm i. d, 2 μm, 100 Å (Thermo Scientific) with a 60 min linear gradient from 3.2 to 40% buffer 0.1% formic acid in © at a flow rate of 300 nl/min. Samples were analyzed using TOP20 HCD; mass data were acquired in a data-dependent strategy, selecting the fragmentation events based on the 20 most abundant precursor ions in the survey scan (375–1,600 Th). The resolution of the survey scan was 60,000 at m/z 200 Th. The ion target values for the survey scans in the Orbitrap and the MS^2^ mode were set to 3E6 and 1E5, respectively, and the maximum injection time was set to 60 ms for both scan modes. The parameters for acquiring HCD MS/MS spectra were set to a collision energy of 27 and an isolation width of 2 m/z. The precursors with unknown charge state or a charge state of 1 were excluded. The peptides selected for MS/MS acquisition were then placed on an exclusion list for 20 s using the dynamic exclusion mode to limit duplicate spectra. Peptides were identified by database searching using Sequest HT and MS Amanda with Proteome Discoverer 2.2 software (Thermo Scientific) against the HBV sequence. Precursor mass tolerance was set at 10 ppm, and fragment mass tolerance was set at 0.02 Da, and up to two missed cleavages were allowed. Oxidation (M), acetylation (protein N-terminus, K) and phosphorylation (S, T, Y) were set as variable modification and carbamidomethylation (C) as fixed modification. Peptides were filtered with a fixed-value PSM validator and rank 1. Phosphorylation sites were then manually validated.

### Circular Dichroism

Far UV circular dichroism (CD) spectra were recorded on a Chirascan spectrometer (Applied Photophysics, Leatherhead, United Kingdom) calibrated with 1S-(+)-10-camphorsulfonic acid. Measurements were carried out at room temperature in a 0.1 cm path length quartz cuvette (Hellma). Spectra were measured in a 180–260 nm wavelength range with an increment of 0.2 nm, band pass of 0.5 nm, and integration time of 1 s. Spectra were processed, baseline-corrected, and smoothed with the Chirascan software. _tag_PreS was in 50 mM phosphate buffer pH 6.5 at a concentration of 9.5 µM.

### Data Availabilitys

The ^13^C and ^15^N backbone chemical shifts of PreS have been deposited in the BioMagResBank (http://www.bmrb.wisc.edu/) under accession code 51186.

## Results

### Cell-free Synthesis and Purification of HBV L and PreS Peptides

**FIGURE 1:**
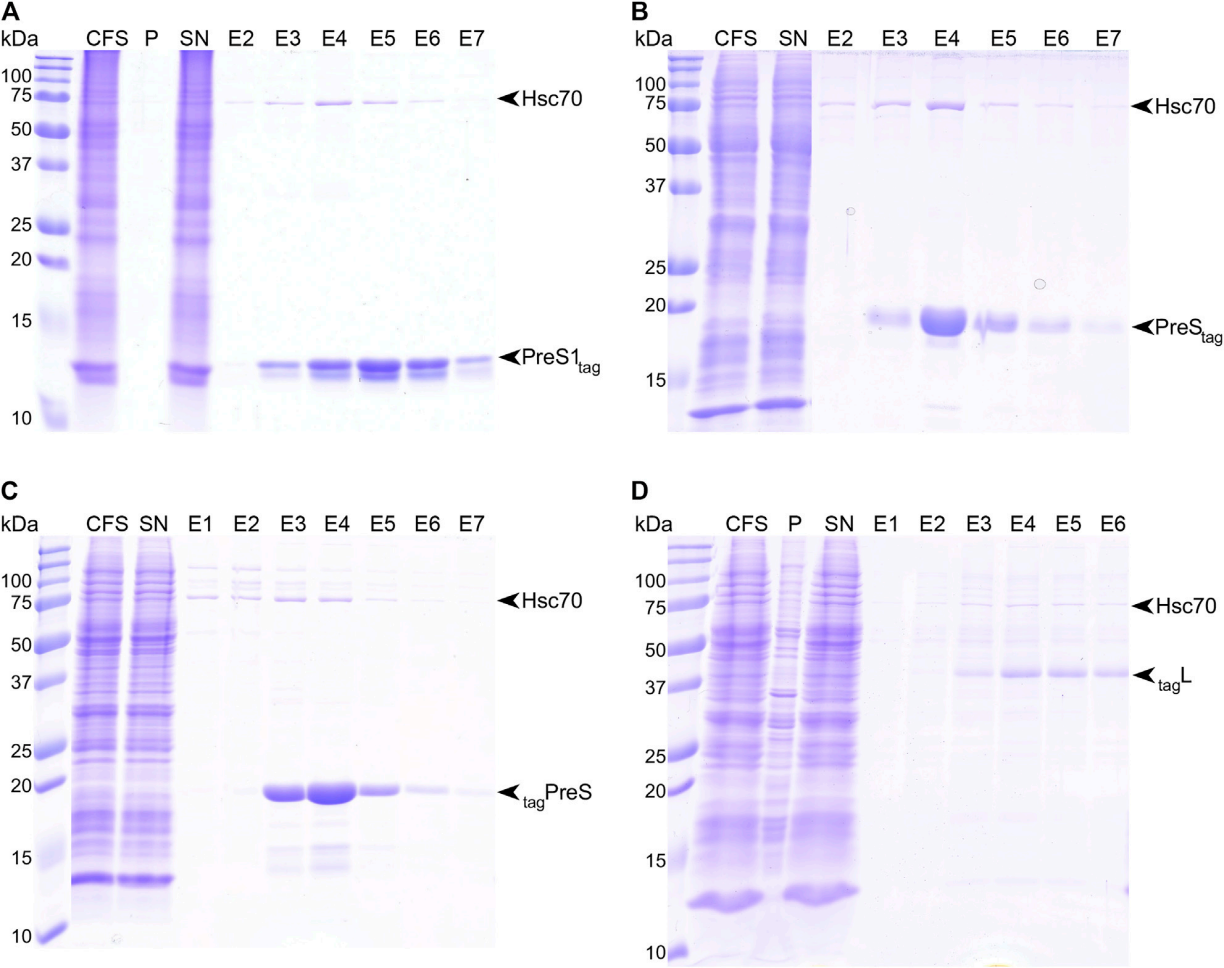
Strep-Tactin© affinity purification of PreS1tag **(A)**, PreS_tag_ **(B)**, _tag_PreS **(C)**, and _tag_L **(D)**. Purification steps have been analyzed by SDS-PAGE, followed by Coomassie blue staining; CFS, total cell-free sample; P, pellet; SN, supernatant; E#, elution fractions.

We synthesized full-length HBV L, as well as PreS1 and PreS fragments thereof, using wheat-germ cell-free protein synthesis (WG-CFPS) (Sawasaki et al., 2002; Takai et al., 2010; Fogeron et al., 2017; Fogeron et al., 2021). All cell-free synthesized proteins carried a Strep-tag II for affinity purification, for L at the N-terminus and for PreS1_tag_ at the C-terminus; we produced both versions for PreS, named _tag_PreS and PreS_tag_. Figure 1A shows the results of the WG-CFPS of PreS1_tag_, followed by affinity purification, as analyzed by SDS-PAGE, followed by Coomassie blue staining. The protein was fully soluble even in the absence of detergent, as no PreS1 was detected in the pellet fraction. The band corresponding to PreS1_tag_ is clearly visible on the gel (yields are given in Supplementary Table S1), and interestingly, two separate bands with different intensities are observed for all elution fractions. We recently reported a similar observation for the duck HBV L protein, where it resulted from alternative translation initiation in addition to phosphorylation (David et al., 2019). Both PreS_tag_ and _tag_PreS have also been successfully purified by affinity chromatography (Figure 1B and Figure 1C, respectively). Final yields (Supplementary Table S1) might be slightly overestimated as some degradation is visible on the gels, indicating that these constructs might not be stable on the long term unless protease inhibitors are present. A CD spectrum was recorded on the purified protein (Supplementary Figure S1), which already indicates that PreS is likely unstructured.

As a membrane protein, the full-length construct _tag_L required the presence of detergent to be synthesized in a solubilized form for further purification. We have identified Brij-58 as a suitable detergent for the soluble production of this construct, with a concentration of 0.05% being optimal. Affinity purification was successfully performed in the presence of 0.1% n-dodecyl-β-D-maltoside (DDM) (Figure 1D). While L_tag_ could be expressed in a soluble form as well, the purification of this construct was unsuccessful, so it was not included in our analyses.

One can note that, for all constructs, in addition to the two bands corresponding to PreS1_tag_, a third band eluted from the column with an apparent molecular weight below 75 kDa, which corresponds to Hsc70, as identified by the mass spectrometry of the band cut from the gel. Hsc70 is known to be an interactant of PreS in cells, and our work thus identifies at least one binding site to be localized in PreS1, in line with previous studies that assigned it to residues 70–107 of PreS (Prange et al., 1999).

In summary, all PreS-containing constructs expressed well using WG-CFPS, with yields between 0.3 and 1.7 mg protein per milliliter of wheat-germ extract (Supplementary Table S1), and in a soluble form, which was obtained for L through addition of detergent to the reaction. All proteins could be purified to high homogeneity, using in some cases detergent, resulting in higher purity.

### Identification of PreS Phosphorylation Sites Using Mass Spectrometry

As multiple bands observed by SDS-PAGE (typically for PreS, Figure 1A) can be an indication of a post-translational modification such as phosphorylation (Ubersax et al., 2003) we set out to assess the modifications using mass spectrometry, in line with previous work on the DHBV L protein (David et al., 2019) and also HBV core (Heger-Stevic et al., 2018). First, the amount of post-translational modifications of PreS1_tag_ were evaluated while analyzing the total mass of the protein by MALDI-TOF mass spectrometry. The region of interest in Supplementary Figure S2 showed four peaks, all corresponding to PreS1_tag_. The first peak from the left corresponds to the protein from which the N-terminal methionine (13,364.0 Da) was stripped. Peaks representing PreS1_tag_ with one additional acetyl group (+42 Da, 13,405.8 Da), with one phosphoryl group (+80 Da, 13,444.2 Da), and with both acetyl and phosphoryl groups (+122 Da, 13,486.3 Da) were observed as well. These signals indicate a single phosphorylation per chain, though not necessarily on the same residue in all proteins in the sample. Major and minor sites can overlap and yield a global sum of one site.

**FIGURE 2:**
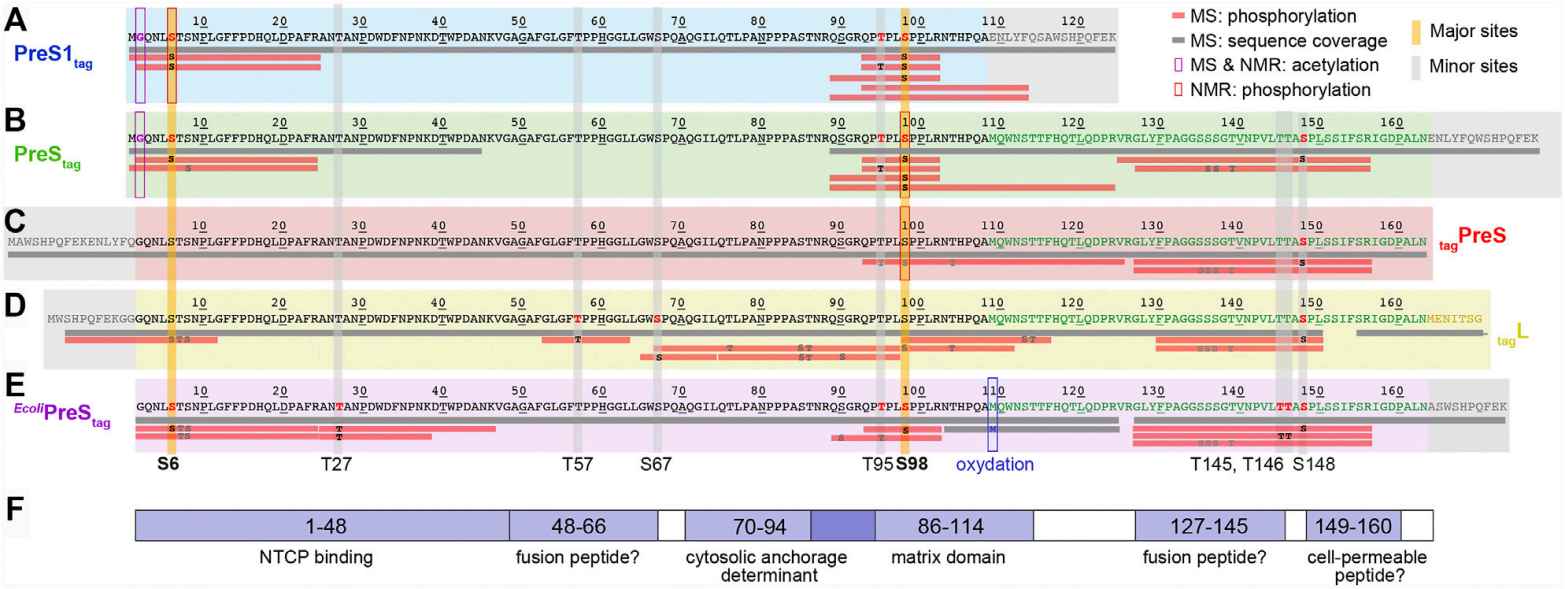
Identification of acetylation and phosphorylation sites in PreS1, PreS, and HBV L using mass spectrometry and NMR. Mass spectrometry analysis has been performed on fractions from purified protein samples shown on Figure 1 for cell-free synthesized PreS1_tag_ **(A)**, PreS_tag_ **(B)**, _tag_PreS **(C)**, _tag_L **(D)**; and MAPK14-^*E. coli*^PreS_tag_ **(E)**. Horizontal gray bars below the sequences represent the sequence coverage by LC-MS/MS. Red bars represent peptides where one or more phosphorylation sites have been confirmed; the residues in black correspond to the phosphorylation sites formally identified, and the ones in gray correspond to possible but not confirmed sites. The PreS1 part of the amino-acid sequence is typed in black, the PreS2 part in green, the beginning of the S part in yellow (the full _tag_L protein was analyzed, but the sequence coverage of S was poor and no phosphorylation sites were detected), and the tag sequences in gray. Additional information extracted from NMR chemical shifts (see below) is shown as purple squares for acetylation and red squares for phosphorylation. Major phosphorylation sites identified by mass spectrometry and NMR are highlighted by vertical yellow bars; minor sites (only mass spectrometry) by vertical gray bars. **(F)** Functional regions of the HBV PreS: binding to NTCP (Yan et al., 2012); possible fusion peptides (Pérez-Vargas et al., 2021); the MD (Bruss and Thomssen, 1994); and a possible cell-permeable peptide (Oess and Hildt, 2000).

In order to localize the modifications on the protein sequence, PreS1_tag_ was further analyzed by LC-MS/MS mass spectrometry (Supplementary Figure S3), and one can see that identified peptides completely covered the sequence of PreS1_tag_ (Figure 2A). Phosphorylation in PreS1_tag_ was unambiguously identified for three amino acids, namely, S6, T95, and S98, which are highlighted in red bold font on the sequence. The analysis also confirmed the removal of M1, as well as acetylation on G2 (Supplementary Figure S4).

For the PreS_tag_ construct, even if the entire sequence was not covered, the analysis by LC-MS/MS mass spectrometry (Figure 2B) revealed phosphorylation on four amino acids: S6, T95, S98, and S148 (in bold red type, Supplementary Figure S5). Further unconfirmed sites pointed to S8, and S136/S137/T139. As for PreS1_tag_, N-terminal methionine processing and G2 acetylation could be identified (data not shown). We also analyzed the complementary construct _tag_PreS, for which LC-MS/MS mass spectrometry shows full sequence coverage (Figure 2C). However, phosphorylation was unambiguously identified only for one single amino acid, namely, S148 (Supplementary Figure S6). Further ambiguous sites are proposed for T95/S98/T104, and also S135/S136/S137/T139. This indicates that the N-terminal tag interferes with phosphorylation in the N-terminal portion.

The LC-MS/MS mass spectra of HBV L (Figure 2D) show that the region between amino acids 50–70 is best covered in L. Three phosphorylation sites could be unambiguously identified in L by LC-MS/MS: T57, S67, and S148 (Supplementary Figure S7). Several ambiguous sites, including S6/T7/S8 and T76/S85/T86, were revealed as well. As the phosphorylation of S6 was clearly identified for the isolated PreS1_tag_ and PreS_tag_ forms as described above, the phosphorylation of HBV L thus most probably also occurs on this residue, and not on T7 or S8.

When combining the results obtained for L and its fragments as produced by WG-CFPS, LC-MS/MS mass spectrometry thus identified S6, T57, S67, T95, S98, and S148 as phosphorylated, and highlights other possible but unconfirmed sites. At the same time, the MALDI-TOF analyses of PreS1 suggested that only one major site exists (S6, T95, S98) and that other sites are minor. These findings are summarized in Figures 2A–D, where horizontal bars represent the peptides that could be analyzed, with those for which phosphorylation was unambiguously confirmed in red.

### Identification of Phosphorylation Sites by NMR Spectroscopy.

**FIGURE 3:**
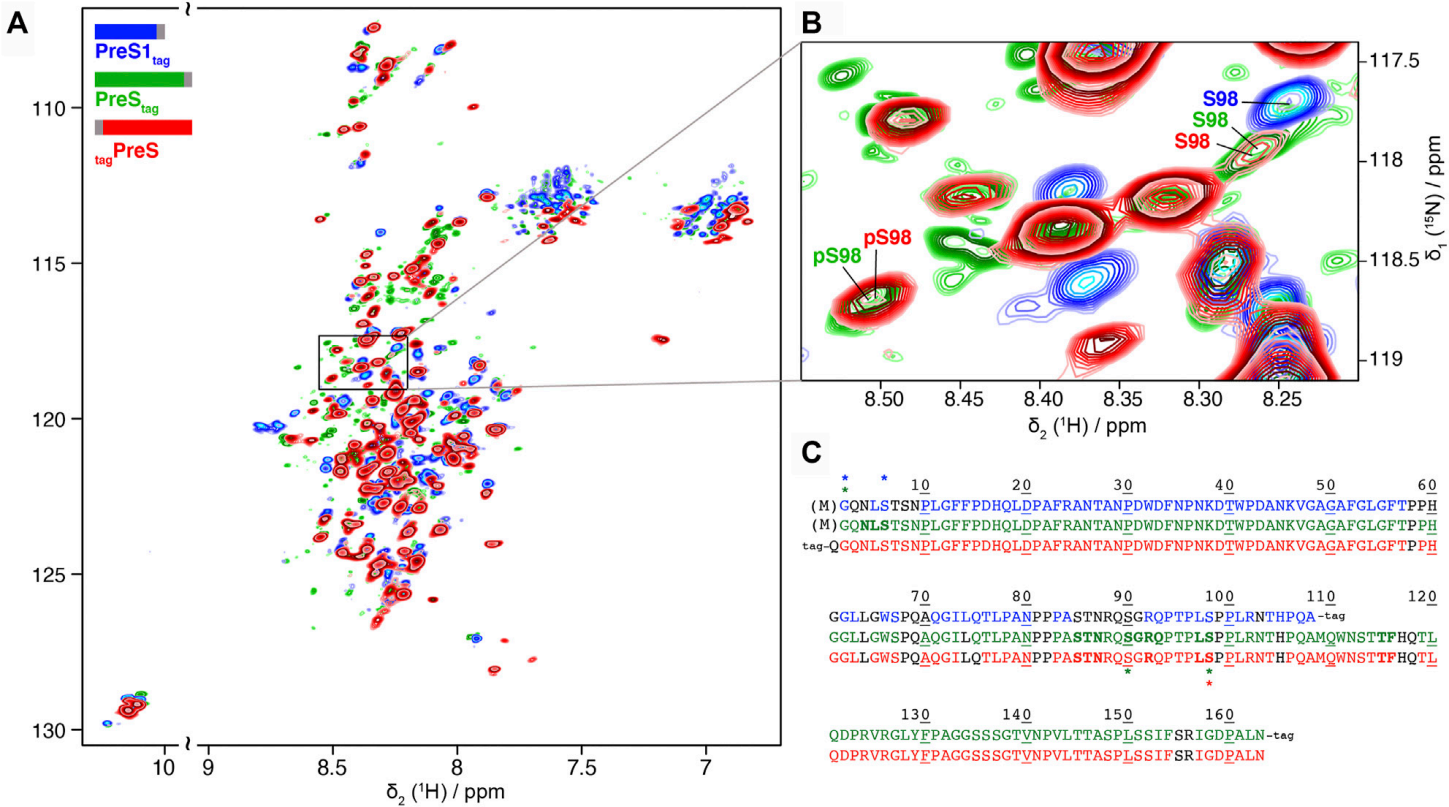
Partial phosphorylation is detected by NMR for PreS and PreS1 produced using cell-free synthesis: **(A)** Solution NMR ^1^H-^15^N BEST-TROSY spectra of ^2^H-^13^C-^15^N PreS1_tag_ at pH 7.5 (in blue), PreS_tag_ at pH 6 (in green) and _tag_PreS at pH 6 (in red). **(B)** Extract of 2D BEST-TROSY spectra showing a peak corresponding to unphosphorylated S98 for the 3 constructs on the top right. The peak is weak for both PreS_tag_ and _tag_PreS, with another peak corresponding to phosphorylated S98 detected at the bottom left. This means that S98 is mainly, but not fully phosphorylated in PreS. The Cβ chemical shift was used to confirm the phosphorylation state of this serine (Supplementary Figure S10). **(C)** Sequence of the three constructs showing assigned residues for PreS1_tag_ (top, blue), PreS_tag_ (middle, green) and _tag_PreS (bottom, red). Assigned residues are colored, while unassigned residues are in black type. Proline residues are colored when their Cα, Cβ and C′ are assigned. Residues which display two forms on the NMR spectra are shown in bold, and residues with chemical-shifts typical of phosphorylation and acetylation are indicated by a star with the corresponding color-code. Processed residue M1 is shown in brackets.

We produced PreS1_tag_, PreS_tag_, and also _tag_PreS on a large scale and uniformly ^2^H-^13^C-^15^N labeled for NMR studies, with yields between 0.3 and 0.6 mg protein per ml WGE (Supplementary Table S1). Solution NMR experiments were recorded on PreS1_tag_, PreS_tag_ and _tag_PreS, and 2D BEST-TROSY spectra are shown in Figure 3A, and an extract in Figure 3B. The peak pattern reveals a narrow chemical shift dispersion, with ^1^H_N_ resonances observed between 7.5 and 8.5 ppm, revealing with atomic detail the intrinsically disordered nature of all three PreS fragments. Backbone resonances were assigned using a combination of 3D NMR spectra (Solyom et al., 2013). Assigned residues are shown on the sequences of the three constructs in Figure 3C, and assignment statistics are given in Supplementary Table S2. A 2D-HN assigned spectrum is shown in Supplementary Figure S8. Secondary chemical shifts derived from the sequential assignments reveal that the proteins do not display any partial secondary structures (Supplementary Figure S9). HBV PreS is thus, as DHBV PreS, an intrinsically disordered protein.

NMR chemical shifts are sensitive to phosphorylation events, as addition of this group typically strongly affects the electronic environment of the neighboring spins. We thus listed random-coil chemical shifts of phosphorylated Ser and Thr residues taken from Hendus-Altenburger et al. (2019) (Supplementary Table S3 and Supplementary Table S4), and compared them to all assigned chemical shifts of Ser and Thr residues in the three samples. This allowed to clearly identify the phosphorylation of S6 in PreS1_tag_, and partial phosphorylation of S98 in both PreS_tag_ and _tag_PreS constructs. S6 and S98 thus correspond to major phosphorylation sites in the WG-CFPS system. S98 is, however, only partially phosphorylated, as can be seen from the presence of a second, weaker signal for this residue at a chemical shift indicative for the non-modified residue, as shown in Figure 3B. The ratios of the peaks allowed to estimate that around 50% of S98 is phosphorylated in PreS_tag_ and 65% in _tag_PreS. The observed peak splitting could possibly also be due to cis–trans isomerization of neighboring proline residues (Hull and Kricheldorf, 1980); still, the chemical shift of the Cβ is typical for a phosphorylated residue (Supplementary Figure S10), and thus clearly points to phosphorylation.

The NMR spectra reveal phosphorylation only for a subset of the residues identified by mass spectrometry as being phosphorylated. This means that several residues, in the different constructs, are only phosphorylated on a subset of proteins in the sample, which is below the detection limit of NMR (about 10%). They thus correspond to minor sites, in agreement with the PreS1_tag_ MALDI-TOF analysis that pointed to a single major phosphorylation site in this construct (Supplementary Figure S1), which thus must be S6 in PreS1_tag_, according to the NMR analysis.

Interestingly, the analysis of the NMR spectra revealed signals corresponding to phosphorylation of an unconfirmed site, S90 in PreS_tag_ (Supplementary Table S3). This site however has not been observed in the two other PreS constructs, and its identity thus remains to be confirmed.

In addition, NMR corroborates removal of the N-terminal methionine and the acetylation of G2 for both PreS1_tag_ and PreS_tag_, as shown in the 3D extracts of the HNcoCAB in Supplementary Figure S11. Indeed, a peak at 24.6 ppm displays a^13^C chemical shift typical of an acetyl glycine, while this is not observed in N-terminally tagged _tag_PreS and _tag_L, where no M1 processing and acetylation can take place due to the tag.

Taken together, the NMR data show that among the phosphorylation sites identified by mass spectrometry, S6 and S98 represent major phosphorylation sites, while T27, T57, S67, T95, T145, T146, and S148 must be minor sites. This is summarized on Figure 2 by the yellow vertical bars, annotated in bold type for major sites, and the gray bars for minor sites.

### Design of an *E. coli* Expression System for Phosphorylated PreS

With major phosphorylation sites being identified according to Figure 2, we predicted, combining information from several web programs (Li et al., 2018; Wang et al., 2020) (http://gps.biocuckoo.org/links.php), the kind of kinase that could generate such a pattern, in order to design a recombinant system to produce phosphorylated PreS in larger amounts for structural and interaction studies. cdk5 and MAPK14 were the best hits, and since cdk5 is reputed to be difficult to produce in bacteria, we included MAPK14 in the *E. coli* co-expression system. The design of the plasmid was based on a previous one developed for the HBV core protein (Heger-Stevic et al., 2018), and included a cleavable GB1 fusion protein in N-terminus. Cleavage results in PreS with a C-terminal *Strep*-tag II as shown in Supplementary Figure S12. We expressed PreS in bacteria with and without MAPK14 co-expression and analyzed the obtained protein with mass spectrometry for phosphorylation. The total mass of the ^*E. coli*^PreS_tag_ co-expressed with MAPK14 was analyzed by MALDI-TOF mass spectrometry, revealing the presence of heterogeneous phosphorylation, with up to five cumulative phosphorylated sites (Supplementary Figure S13). The identified phosphorylation sites by LC-MS/MS mass spectrometry are shown in Figure 2E, with confirmed sites at S6, T27, T95, S98, T145, T146, and S148 (Supplementary Figure S14), with T95 possibly also assigned to S90. Also, similarly as for L_tag_, T7 and S8 have been proposed as alternatives to S6; yet with S6 clearly confirmed for PreS1_tag_ and PreS_tag_, T7 and S8 are unlikely options. Four sites overlap with the previously identified phosphorylation sites using WG-CFPS, namely, S6, T95, S98, and S148. Surprisingly, while mass spectrometry allowed to evidence phosphorylation in ^*E. coli*^PreS_tag_, it could not be detected by NMR as shown in Supplementary Figure S15. Most likely, phosphorylation in the current recombinant system is not quantitative as seen in the mass spectrum from Supplementary Figure S13 where the major species remains the unphosphorylated protein, which probably places the phosphorylated residues below the detection level of 2D and 3D NMR spectra. This highlights the interesting ability of the cell-free system to efficiently induce phosphorylation by endogenous kinases.

## Discussion

We have shown that the HBV L protein is phosphorylated at several sites *in vitro* when synthesized both in a wheat-germ cell-free system or by bacterial co-expression with MAPK14. Proteomics studies (Mak et al., 2006) on wheat germs have highlighted the presence of several kinases, including also serine/threonine kinases; a study of kinases present in different organs of the wheat plant has revealed that germs contain a variety of kinases as well (Wei and Li, 2019). The phosphorylation of DHBV L in WG-CFPS has been observed to mainly occur at sites, followed by a proline residue, however, with exceptions (David et al., 2019). This is also the case for HBV L, where five sites are followed by Pro. Interestingly, as also for DHBV L, the N-terminal site (S6 in HBV L and S8 in DHBV L, respectively) is not preceding a Pro. Most residues for which phosphorylation could be confirmed are highly conserved in the sequence, and also most subsequent Pro residues (with the exception of 149), as shown in Supplementary Figure S15. An exception is S27, which is in about 20% of cases a Thr. We identified a total of nine phosphorylation sites, with several observed in different constructs by mass spectrometry and two confirmed by NMR as being major.

All identified sites are located in or just next to regions closely linked to different PreS functions [reviewed recently in Sun et al. (2021)] as shown in Figure 2F. Indeed, S6 is located in the interaction sequence of L with the cellular HBV receptor NTCP (Gripon et al., 2005; Yan et al., 2012). Since S6 is close to the central myristoylation site, its phosphorylation clearly has the potential to impact NTCP binding. T27 is located inside the identified NTCP-binding peptide as well. T57, S67, and T145 are located in or just next to the putative fusion peptides that have been proposed recently using a combined computational and experimental approach (Pérez-Vargas et al., 2021). Residues A70–P94 have been described to contain the amino-acid stretch that determines the cytosolic anchorage of PreS, presumably through interaction with the cognate heat shock protein Hsc70 (Prange et al., 1999). T95 and S98 are located in the so-called matrix domain (MD), which comprises the stretch of amino acids T86–T114 in the large envelope protein L. MD is the presumed interaction site of PreS with the core protein of the HBV capsid, central in the process of envelopment (Poisson et al., 1997; Le Pogam and Shih, 2002). This region is crucial for virion formation, and is believed to establish contact to the nucleocapsid, since truncations up to G92 were compatible with envelopment (Bruss and Thomssen, 1994), and several point mutations within the MD-blocked virion formation (Bruss, 1997). It has been shown also that a peptide comprising this domain interacts with the core particle (Poisson et al., 1997). Phosphorylation on T95 and S98 is centrally located in this domain and has the potential to change the required interaction interfaces, resulting in productive envelopment. S148 is located just downstream from the proposed amphipathic PreS2 translocation motif (Oess and Hildt, 2000; Sun et al., 2021). It is intriguing that in, or just next to, each proposed functional sites, phosphorylation is observed in the present experiments, pointing to a possible role thereof in the regulation of PreS function. No role for phosphorylation has yet been identified for the phosphorylation sites in DHBV PreS (Grgacic and Anderson, 1994; Grgacic et al., 1998). However, DHBV differs from HBV in fundamental aspects, including by a much-large core protein (Makbul et al., 2020) and the lack of an HBx-like transactivator that is crucial in HBV infection (Slagle and Bouchard, 2018). Hence, a functional relevance of PreS phosphorylation in the human virus would not be surprising.

## Conclusion

We here reported phosphorylation on nine different sites of the PreS domain of the human HBV L envelope protein, enabled by endogenous kinases in the wheat germ extract used for cell-free protein synthesis, or in *E. coli* by co-expression with the MAPK14 kinase. We identified two major phosphorylation sites, on S6 and S98, and seven minor ones, using a combination of mass spectrometry and NMR. We found phosphorylation to occur in all major functional regions of PreS, which raises the possibility that phosphorylation is involved in the regulation of these functions in the human virus protein, although this has not been identified in the avian virus homolog. We however found also that full phosphorylation is provided in neither recombinant system, which positions phosphorylation mimics by S/T to E mutations as the best strategy to explore the impact of phosphorylation on preS interactions in structural studies.

## Data Availability

The datasets presented in this study can be found in online repositories. The names of the repository/repositories and accession number(s) can be found below: https://bmrb.io, 51186.
