# Supplementary material for: Phosphorylation of the Hepatitis B Virus Large Envelope Protein
Source: Front Mol Biosci. 2022 Feb 23;8:821755. doi: 10.3389/fmolb.2021.821755 (PMC8904964; doi:10.3389/fmolb.2021.821755)
Supplement: Supplementary file 1 [file DataSheet1.PDF]

SUPPLEMENTARY MATERIAL FOR:

Phosphorylation of the hepatitis B virus large envelope protein

Marie-Laure Fogeron[1#], Lauriane Lecoq[1#], Laura Cole[1], Roland Montserret[1], Guillaume David[1], Adeline Page[2], Frédéric Delolme[2], Michael Nassal[3], Anja Böckmann[1]

[1]*Molecular Microbiology and Structural Biochemistry, Labex Ecofect, UMR 5086 CNRS/Université de Lyon, 69367 Lyon, France*

[2] *Protein Science Facility, SFR BioSciences CNRS UAR 3444, Inserm US8, UCBL, ENS de Lyon, 50 Avenue Tony Garnier, 69007 Lyon, France*

[3]*University Hospital Freiburg, Dept. of Medicine II / Molecular Biology, Medical Center, University of Freiburg, Germany*

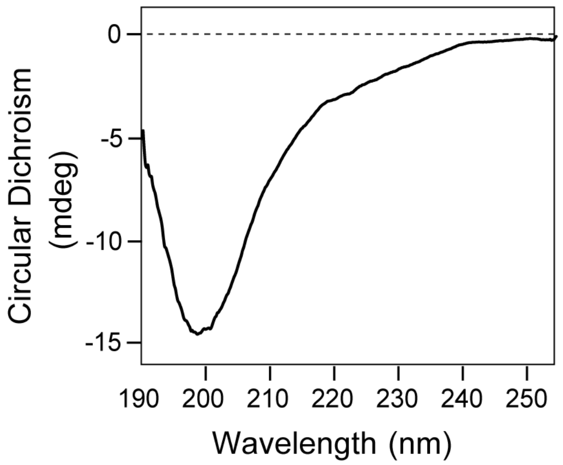

Figure S1. Far UV CD spectrum of wheat-germ cell-free protein synthesis (WG-CFPS) $_{tag}$PreS after affinity chromatography purification. A large negative peak around 200 nm and the absence of any other peak are the indicative of an intrinsically unstructured protein.

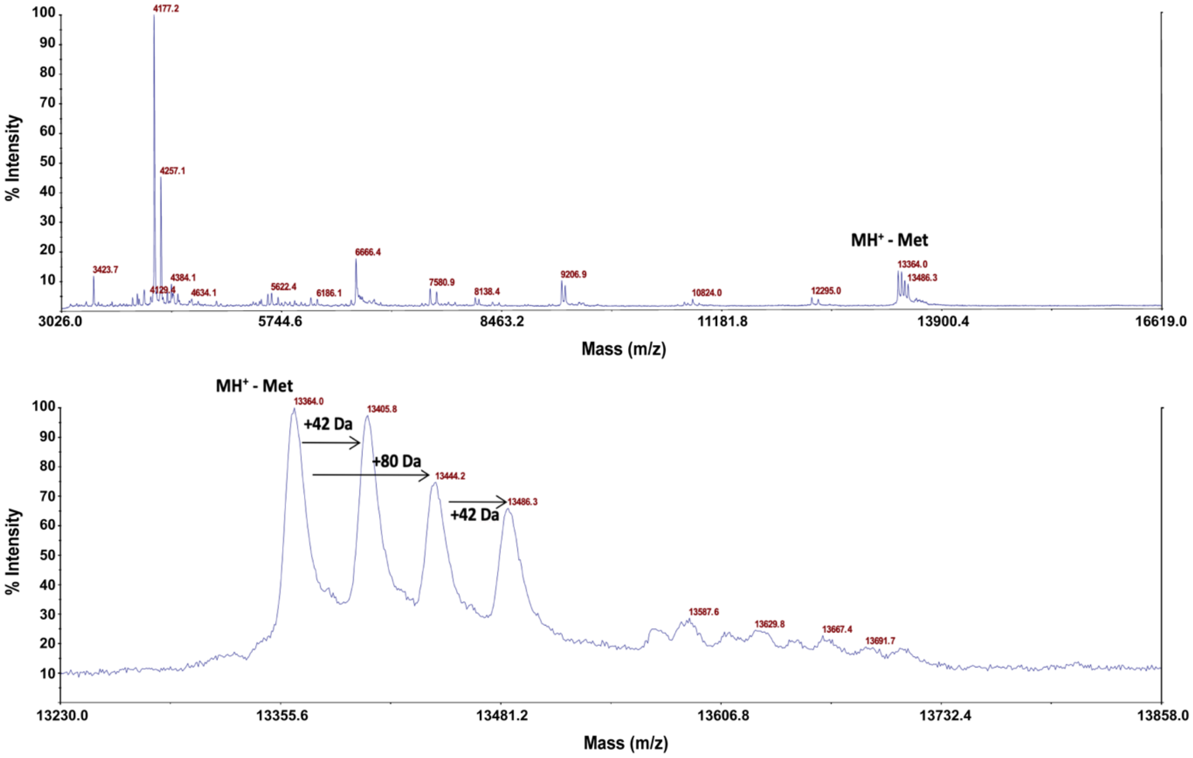

Figure S2. MALDI-TOF Mass spectrometry analysis of WG-CFPS PreS1$_{tag}$ domain. The full spectrum (top) and the zoom (bottom) correspond to the analysis of the purified protein as shown in Figure 1a. The first peak from the left corresponds to the protein from which the N-terminal methionine (13364.0 Da) was stripped. The additional peaks at +42 Da and +80 Da correspond to an acetylation and a phosphorylation, respectively.

Ser6

Peptide [2-24] : MH$^+$: 2638.2151 Th - **MSMS on** m/z 880.0765 (3+ ; 2.45 ppm)

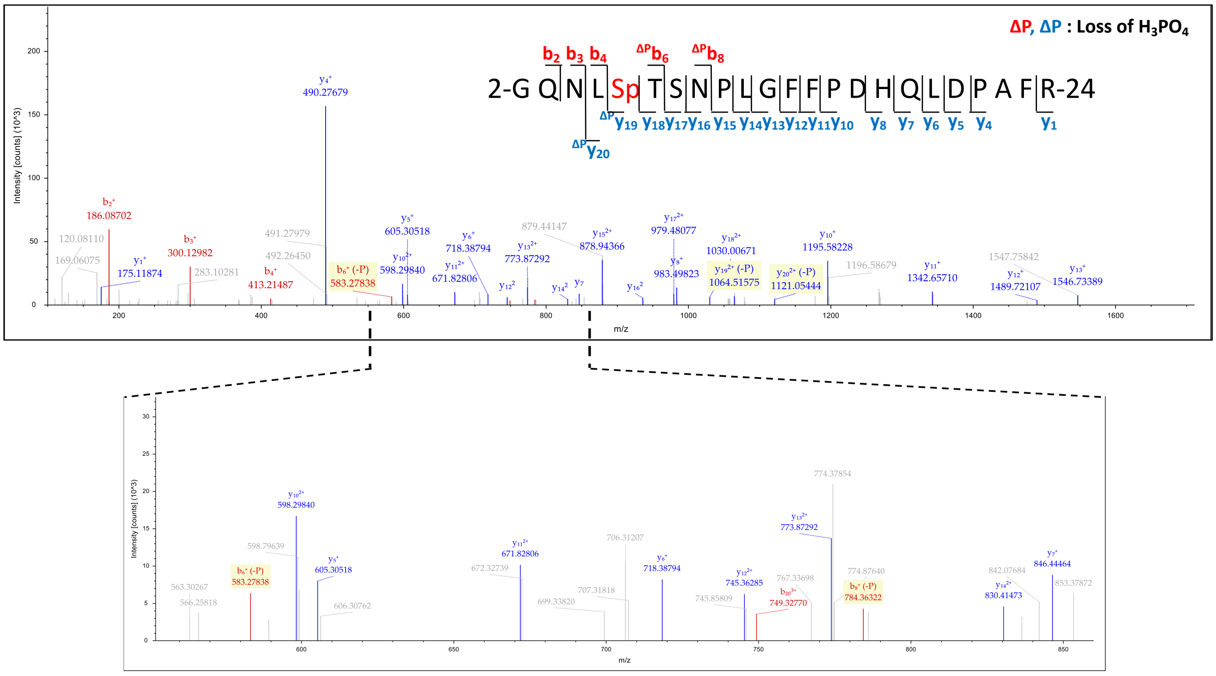

Thr95

Peptide [93-102] : MH$^+$: 1185.6036 Th - **MSMS on** m/z 593.3054 (2+ ; 0.69 ppm)

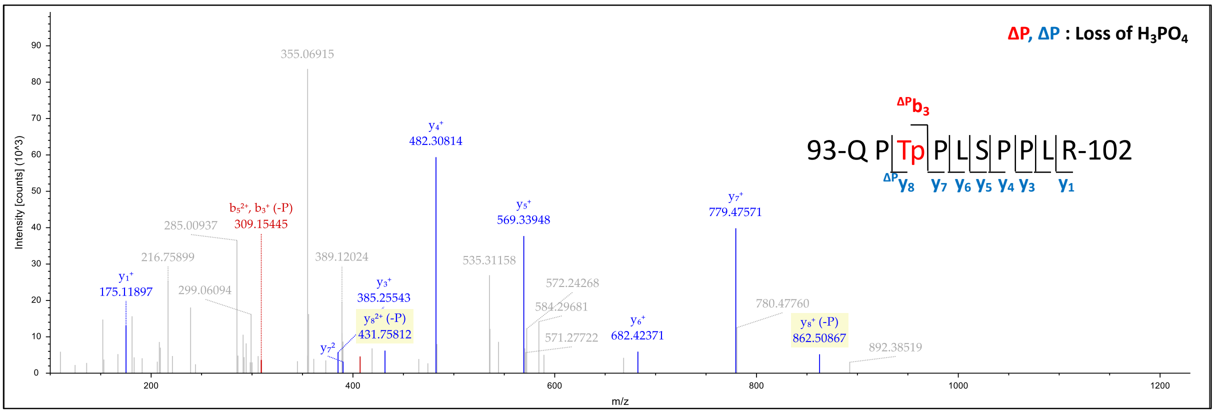

Ser98

Peptide [93-102] : MH⁺ : 1185.6050 Th - **MSMS on** m/z 593.3061 (2+ ; 1.92 ppm)

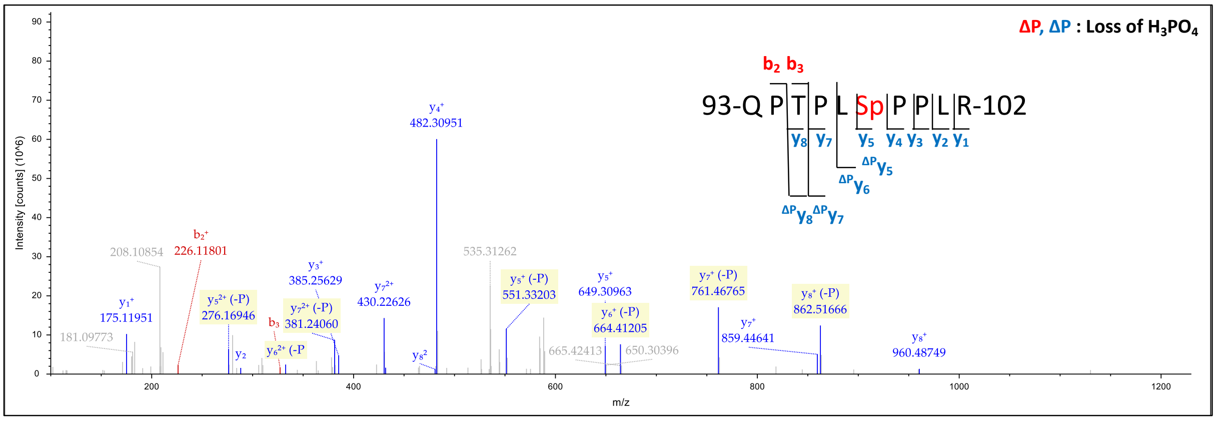

Figure S3. LC-MS/MS Mass spectrometry analysis identifying phosphorylation sites of WG-CFPS PreS1$_{tag}$. Shown spectra correspond to the analysis of the upper band of the purification as shown in Figure 1a, the confirmed phosphorylation sites are indicated in red for residues S6, T95 and S98.

Gly2

Peptide [2-24] : MH⁺ : 2600.2547 Th - **MSMS on** m/z 867.4231 (3+ ; 0.73 ppm)

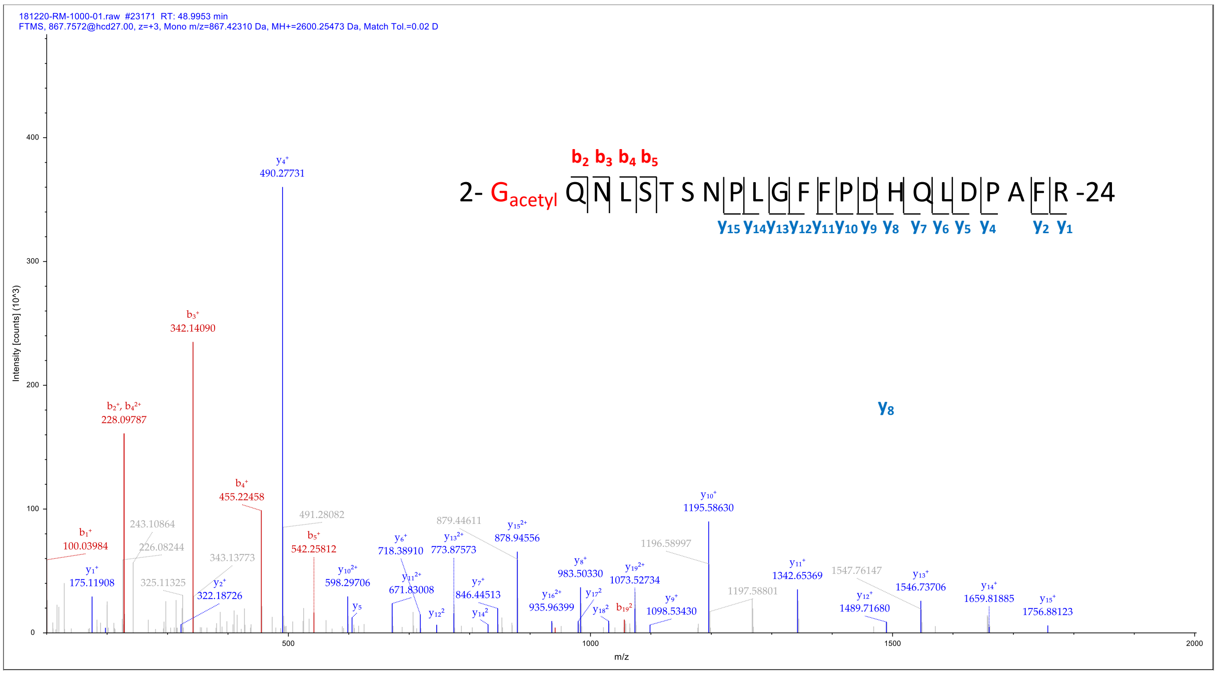

Figure S4. LC-MS/MS Mass spectrometry analysis identifying acetylation of WG-CFPS PreS1$_{tag}$. The shown spectrum confirms processing of M1 as well as the N-terminal acetylation of G2.

Ser6

Peptide [2-24] : MH$^+$: 2638.2055 Th - **MSMS on** m/z 880.0734 (3+ ; -1.16 ppm)

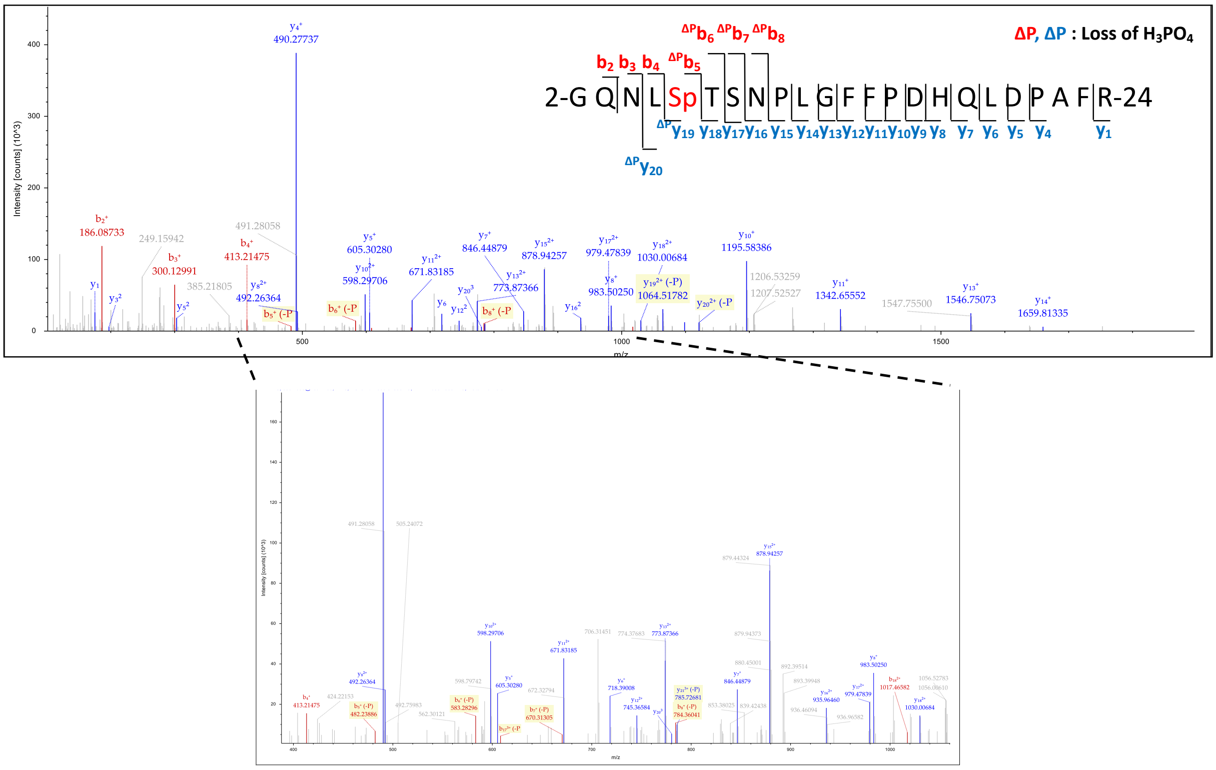

Thr95

Peptide [93-102] : MH$^+$: 1185.6034 Th - **MSMS on** m/z 593.3054 (2+ ; 0.58 ppm)

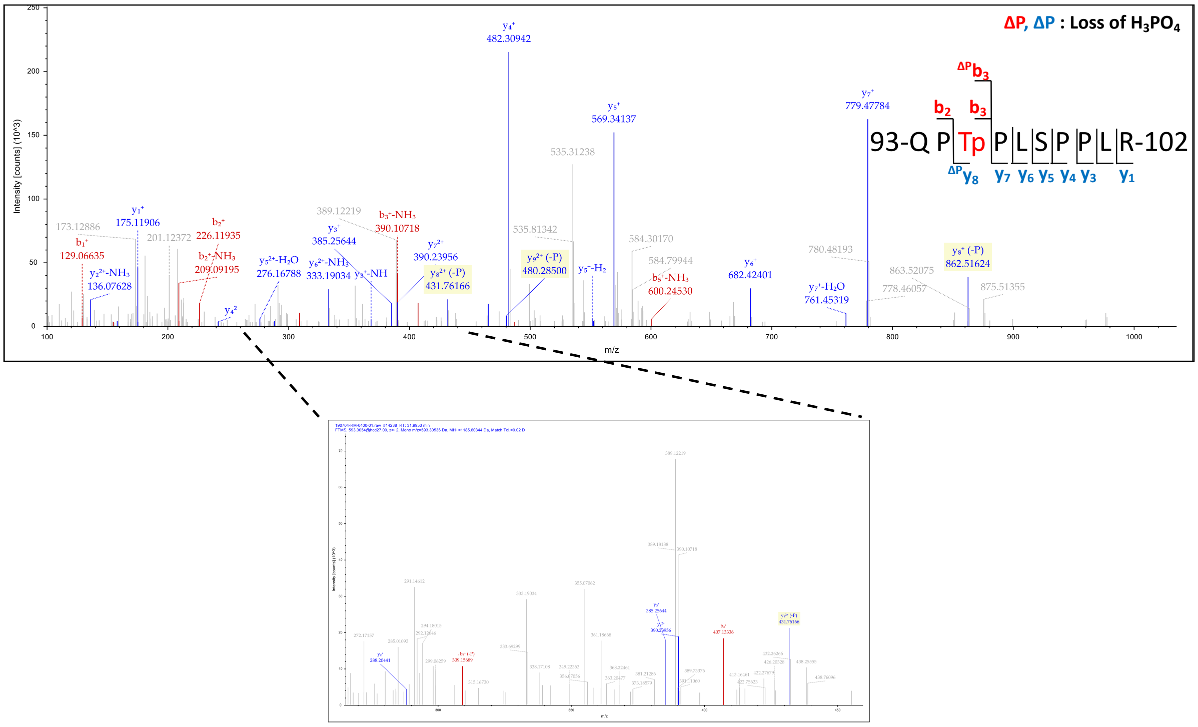

Ser98

Peptide [93-102] : MH⁺ : 1185.6021 Th - **MSMS on** m/z 593.3047 (2+ ; -0.55 ppm)

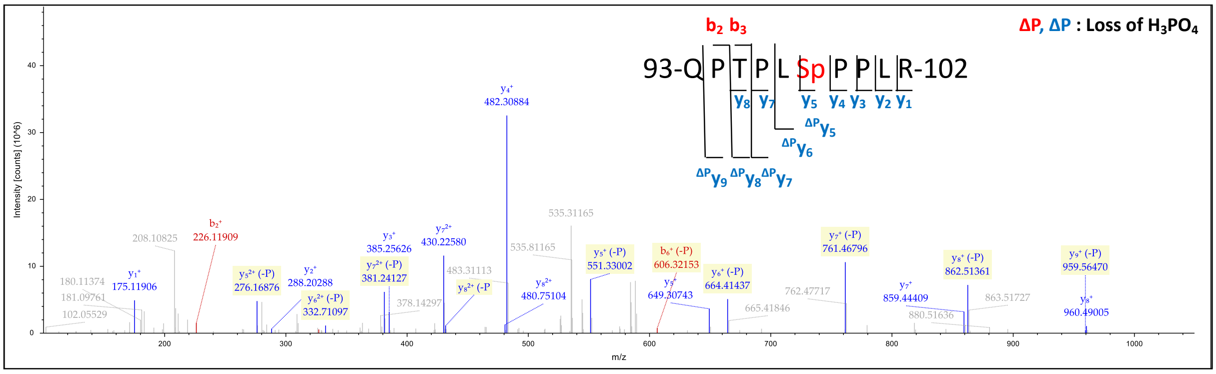

Ser148

Peptide [127-156] : MH⁺ : 3050.47490 Th - **MSMS on** m/z 1525.47490 (2+ ; -3.99 ppm)

ΔP, ΔP : Loss of H_3PO_4

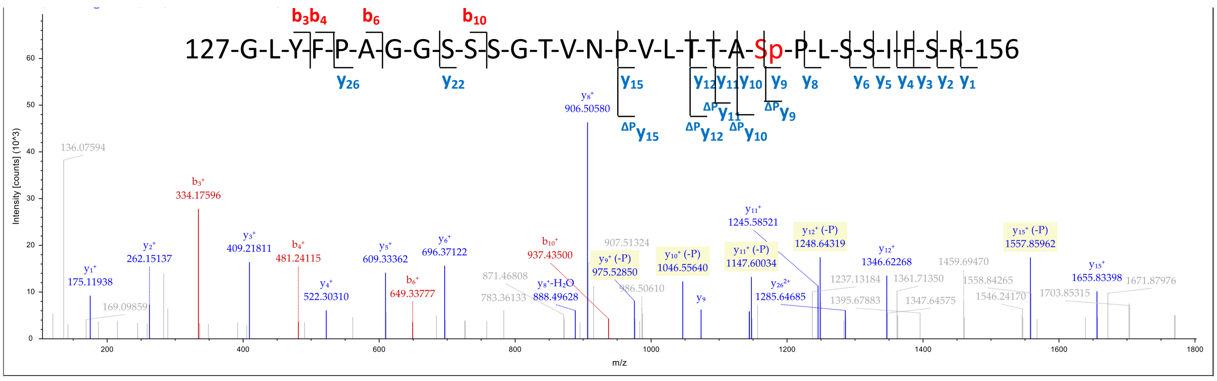

Figure S5. LC-MS/MS Mass spectrometry analysis identifying phosphorylation sites of WG-CFPS PreS$_{tag}$. Shown spectra correspond to the analysis of the band of the purification as shown in Figure 1b, the identified phosphorylation sites are indicated in red for residues S6, T95 S98 and S148.

Ser148

Peptide [127-157] : MH⁺ : 3050. 45928 Th - **MSMS on** m/z 1525.73328 (2+ ; -9.11 ppm)

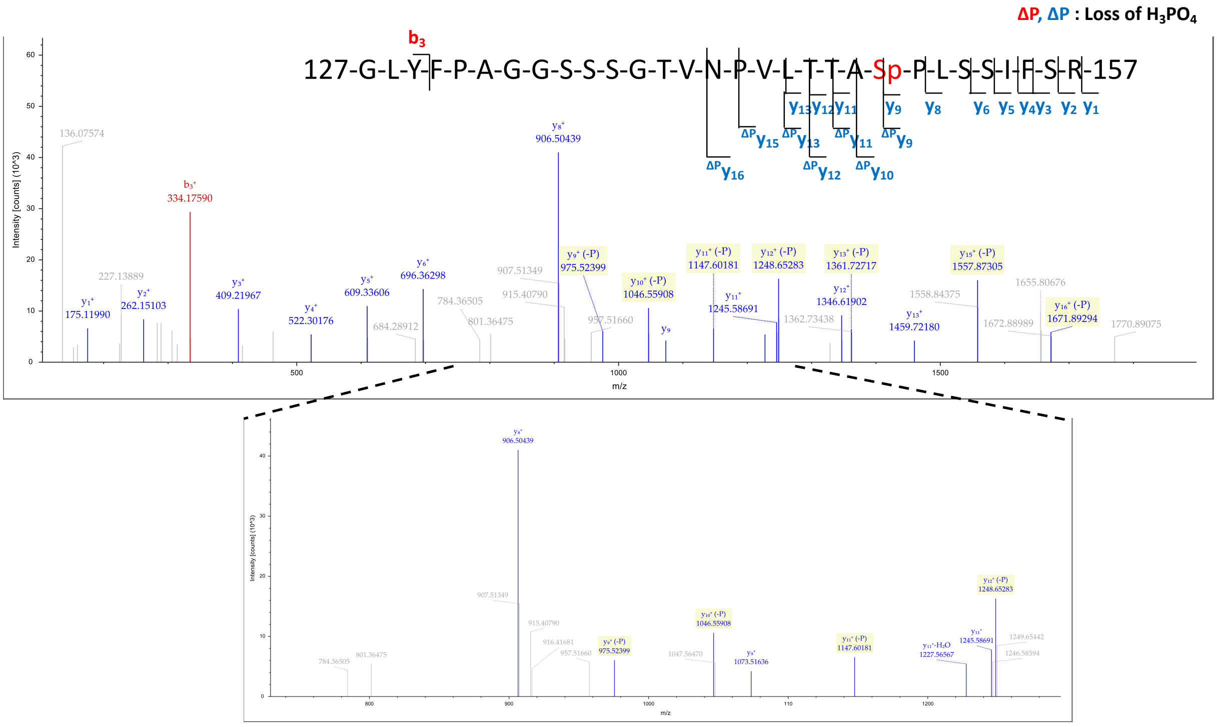

Figure S6. LC-MS/MS Mass spectrometry analysis identifying a phosphorylation site of WG-CFPS $_{tag}$PreS. Shown spectra correspond to the analysis of the band of the purification as shown in Figure 1c, the confirmed phosphorylation site is indicated in red for residue S148.

Thr57

Peptide [57-63] : MH⁺ : 1132.5186 Th - **MSMS on** m/z 566.76294 (2+ ; -0.07 ppm)

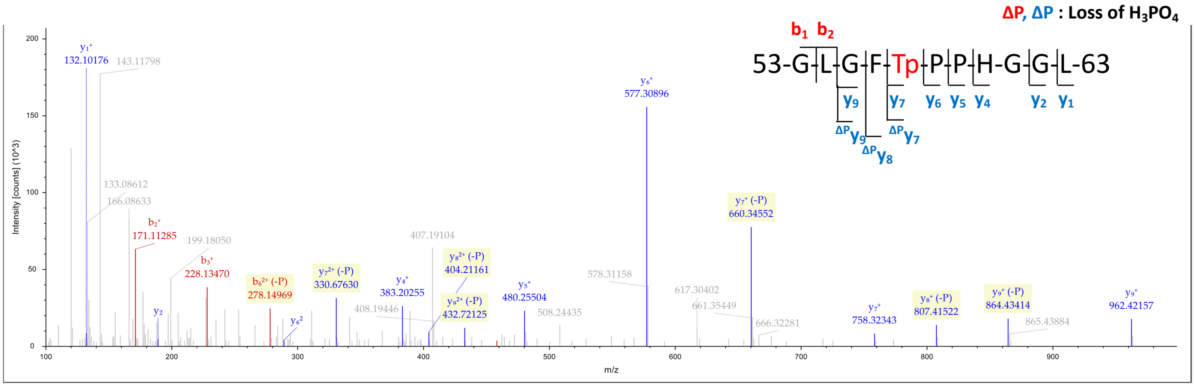

Ser67

Peptide [76-85] : MH⁺ : 1136.51409 Th - **MSMS on** m/z 568.76068 (2+ ; 0.43ppm)

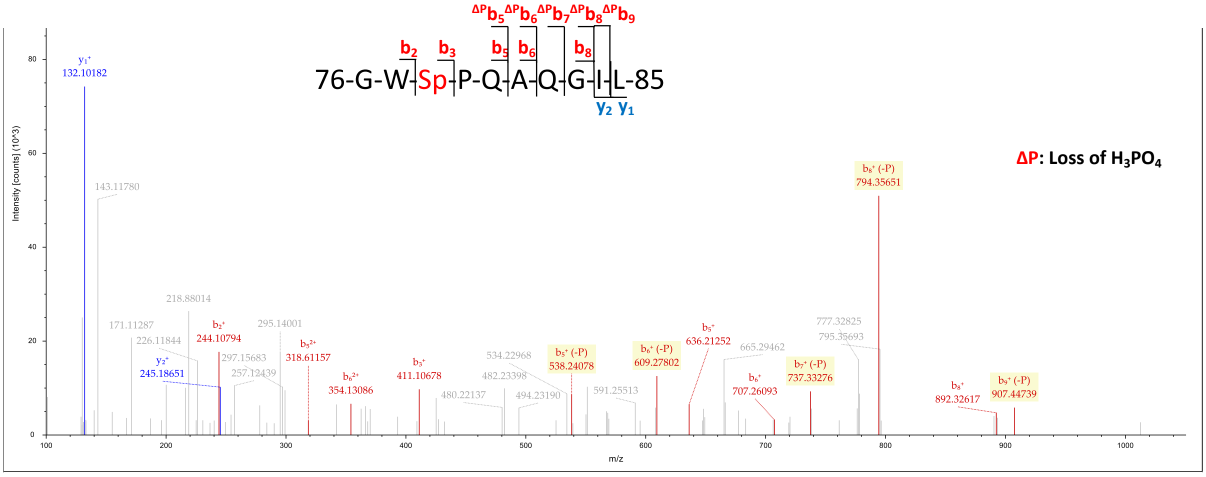

Ser148

Peptide [130-150] : MH⁺ : 2039.95732 Th - **MSMS on** m/z 1020.4823 (2+ ; -5.49 ppm)

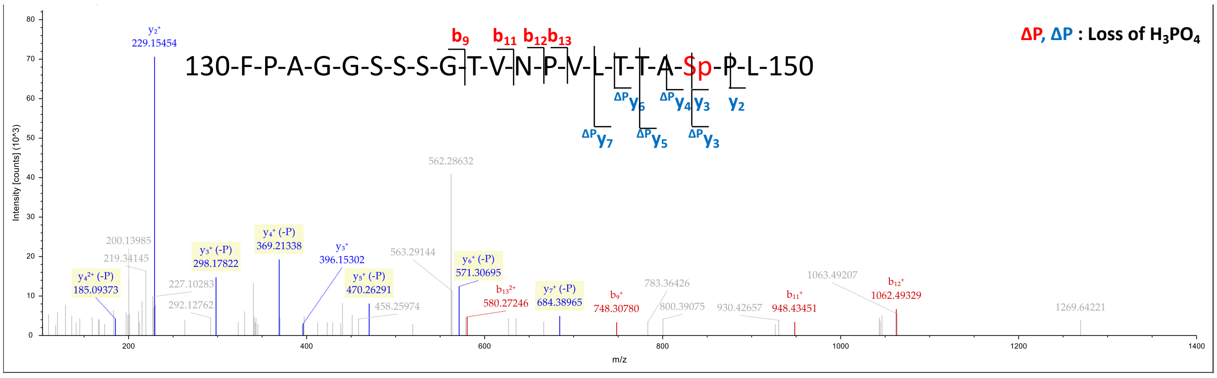

Figure S7. LC-MS/MS Mass spectrometry analysis identifying phosphorylation sites of WG-CFPS $_{tag}$L. Shown spectra correspond to the analysis of the band of the purification as shown in Figure 1d, the confirmed phosphorylation sites are indicated in red for residues T57, S67 and S148.

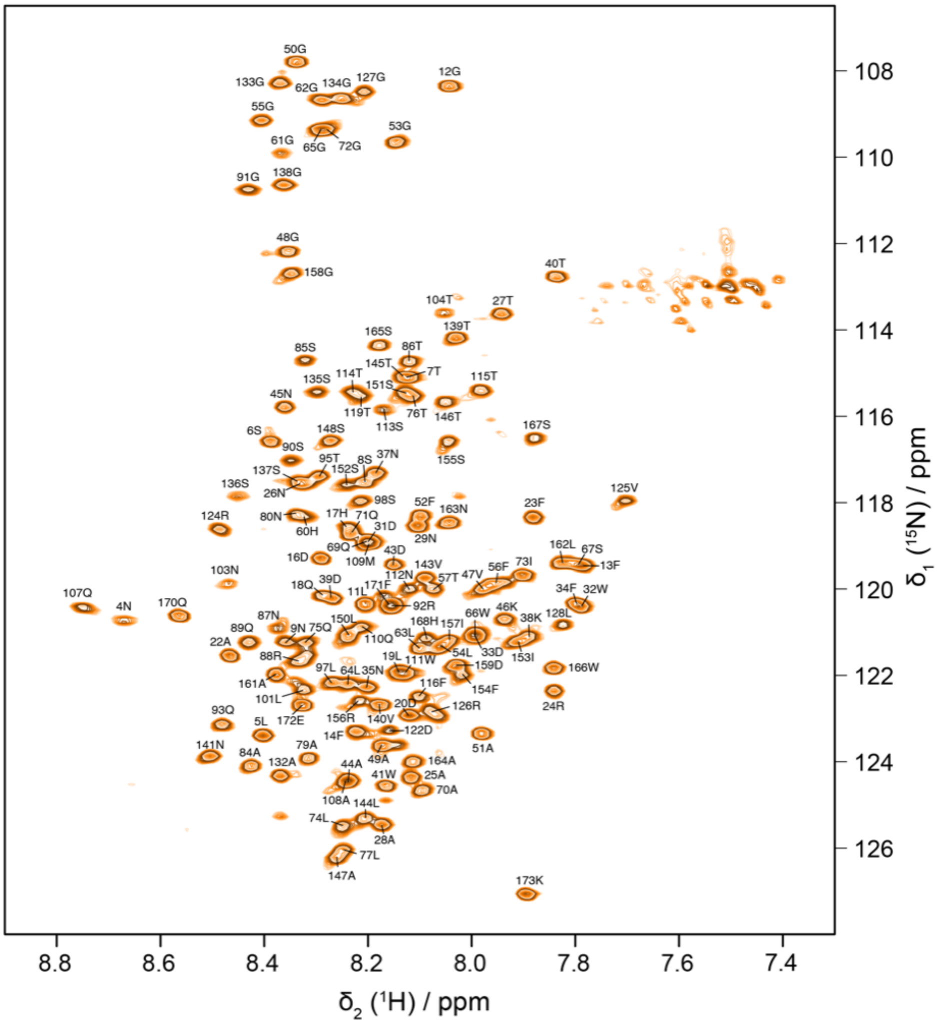

Figure S8. Assigned HN-BTROSY spectrum of $^{E.coli}$PreS$_{tag}$. Assignment is labeled on the amide region of a best-trosy-HN spectrum of the $^{E.coli}$PreS$_{tag}$ sample in 20 mM NaPO4 pH 6, 50 mM NaCl, 7 % D2O concentrated at 50 µM. The spectrum was recorded at 298 K on a 600 MHz spectrometer equipped with a cryoprobe for 2h17 with 32 scans.

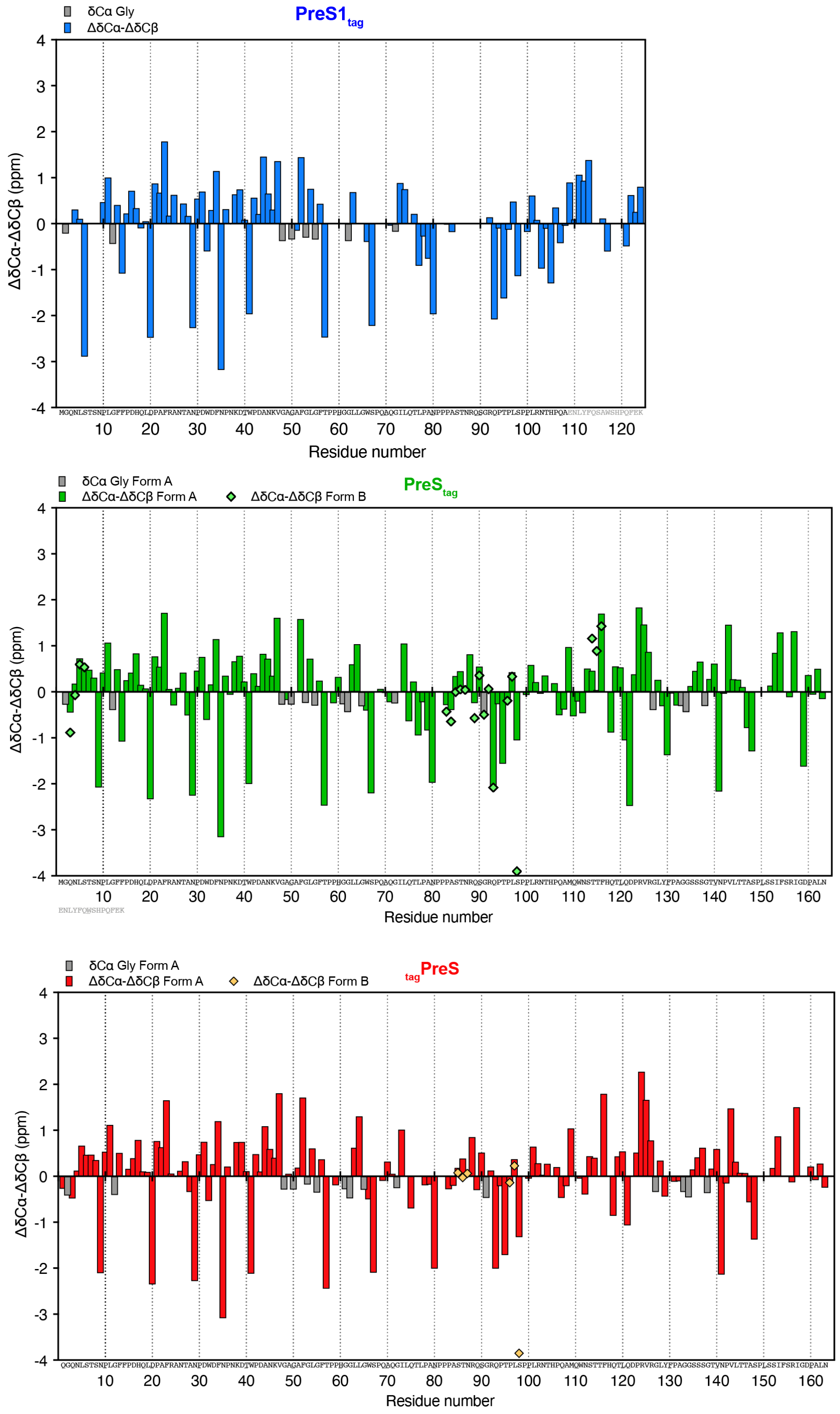

Figure S9. Differences between secondary chemical shifts of Cα and Cβ resonances relative to their random coil shift (Wang and Jardetzky, 2002) as a function of residue number for WG-CFPS PreS1 and both PreS constructs. For glycine residues (shown as grey bar), the deviation of the Cα shift from the random-coil value is plotted.

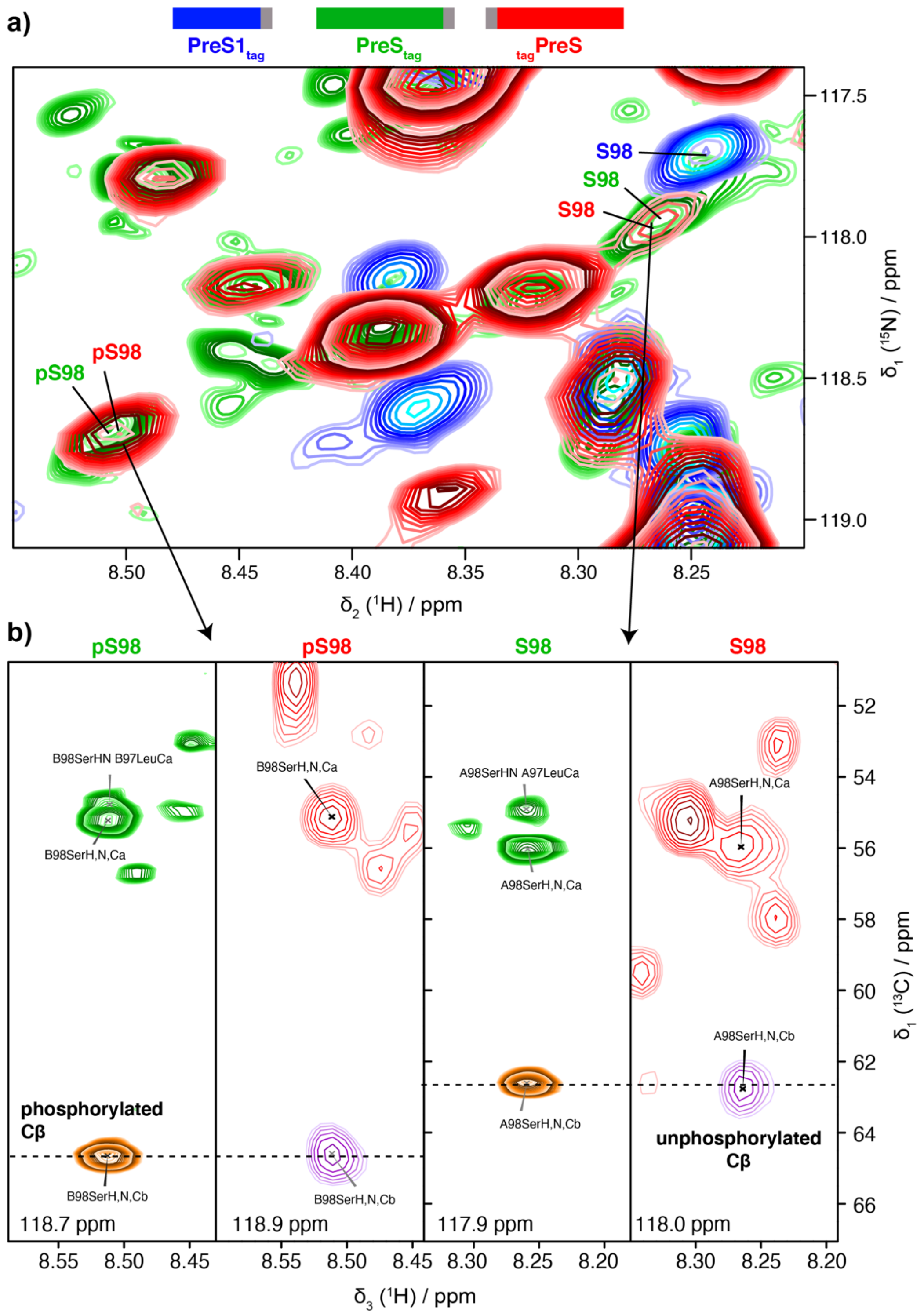

Figure S10. Chemical shifts of S98 indicate a partial phosphorylation in WG-CFPS PreS. a) Extract of 2D BEST-TROSY as in Figure 3 showing 2 forms for S98 in $PreS_{tag}$ (green) and $_{tag}PreS$ (red) but only one in $PreS1_{tag}$ (blue). **b)** 2D planes extracted from 3D HNCACB of S98 in $PreS_{tag}$ and $_{tag}PreS$. Positive signals are shown respectively in green and red (Cα), and negative signals in orange and purple (Cβ). The two left panels show Cβ chemical shift typical of phosphorylated serine, while the two right panels show the unphosphorylated S98 (see Table S3).

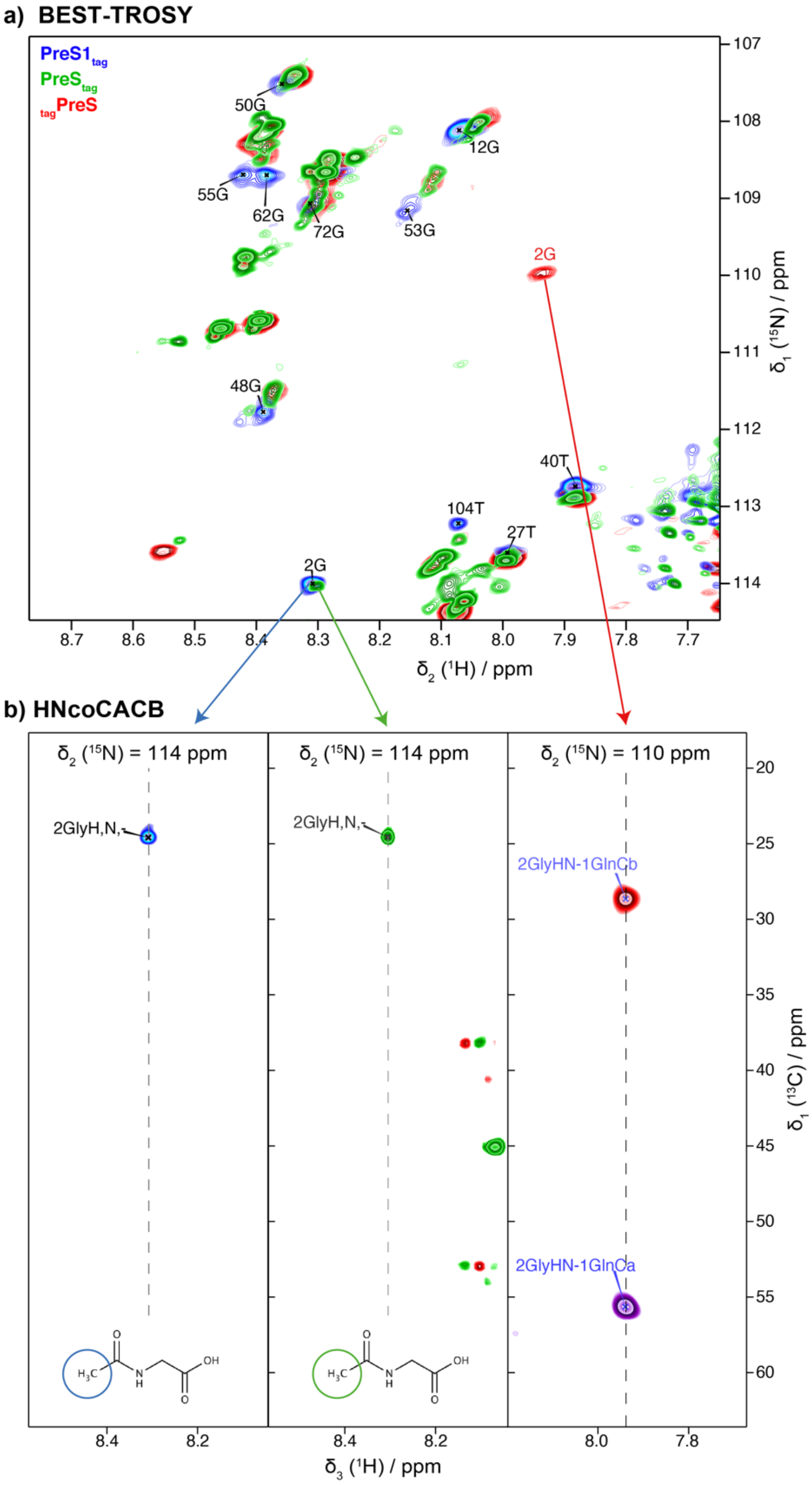

Figure S11. Gly2 is acetylated in WG-CFPS PreS1$_{tag}$ and PreS$_{tag}$. a) Extract of 2D BEST-TROSY on the glycine region. Shifted G2 overlays well for PreS1$_{tag}$ (blue) and PreS$_{tag}$ (green), while it is found in the typical glycine region for $_{tag}$PreS (red). **b)** 2D planes extracted from 3D HNcoCACB, which show signals for PreS1$_{tag}$ and PreS$_{tag}$ at 24.6 ppm, which match the chemical shift of an acetyl group (inserts). For $_{tag}$PreS, the Cα and Cβ resonances from preceding residue Q1 (from the tag) are visible.

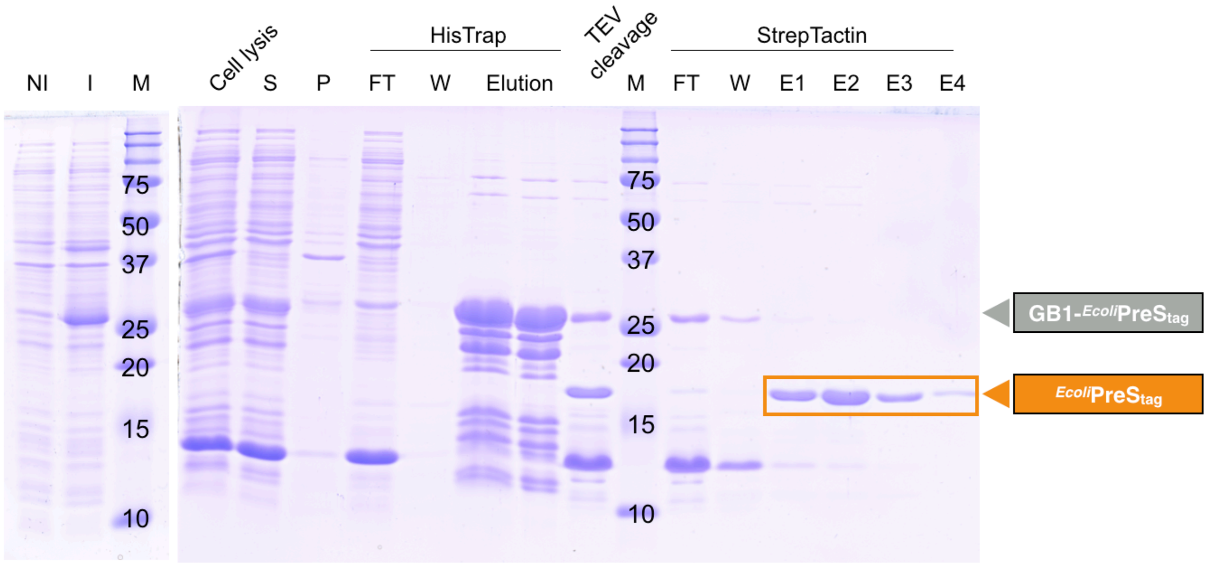

Figure S12. SDS-PAGE (15 % acrylamide) of $^{E.coli}$PreS$_{tag}$ expression and purification. NI: non-induced; I: induced; M: molecular weight marker; S: supernatant: P: pellet; FT: flow-through; W: Wash; E1-E4: elution fractions. The fusion protein GB1-^{Ecoli}PreS$_{tag}$ (grey arrow, ~27 kDa) is well expressed as seen in the induced fraction (I), and is mostly found in the soluble fraction (S) after cell lysis. The supernatant was loaded on a HisTrap column where GB1-^{Ecoli}PreS$_{tag}$ attaches *via* its His-tag in N-ter. After elution, TEV protease is used to cleave GB1 from PreS, and three distinct bands can be seen on the 'TEV cleavage' lane: TEV protease (27,8 kDa), ^{Ecoli}PreS$_{Tag}$ (18,4 kDa, orange arrow) and GB1 (8,7 kDa). The solution is then loaded on a StrepTactin column where ^{Ecoli}PreS$_{tag}$ binds *via* its Streptag. After washing the column, the final sample is eluted (E1-E4), resulting in pure ^{Ecoli}PreS$_{tag}$ (orange square).

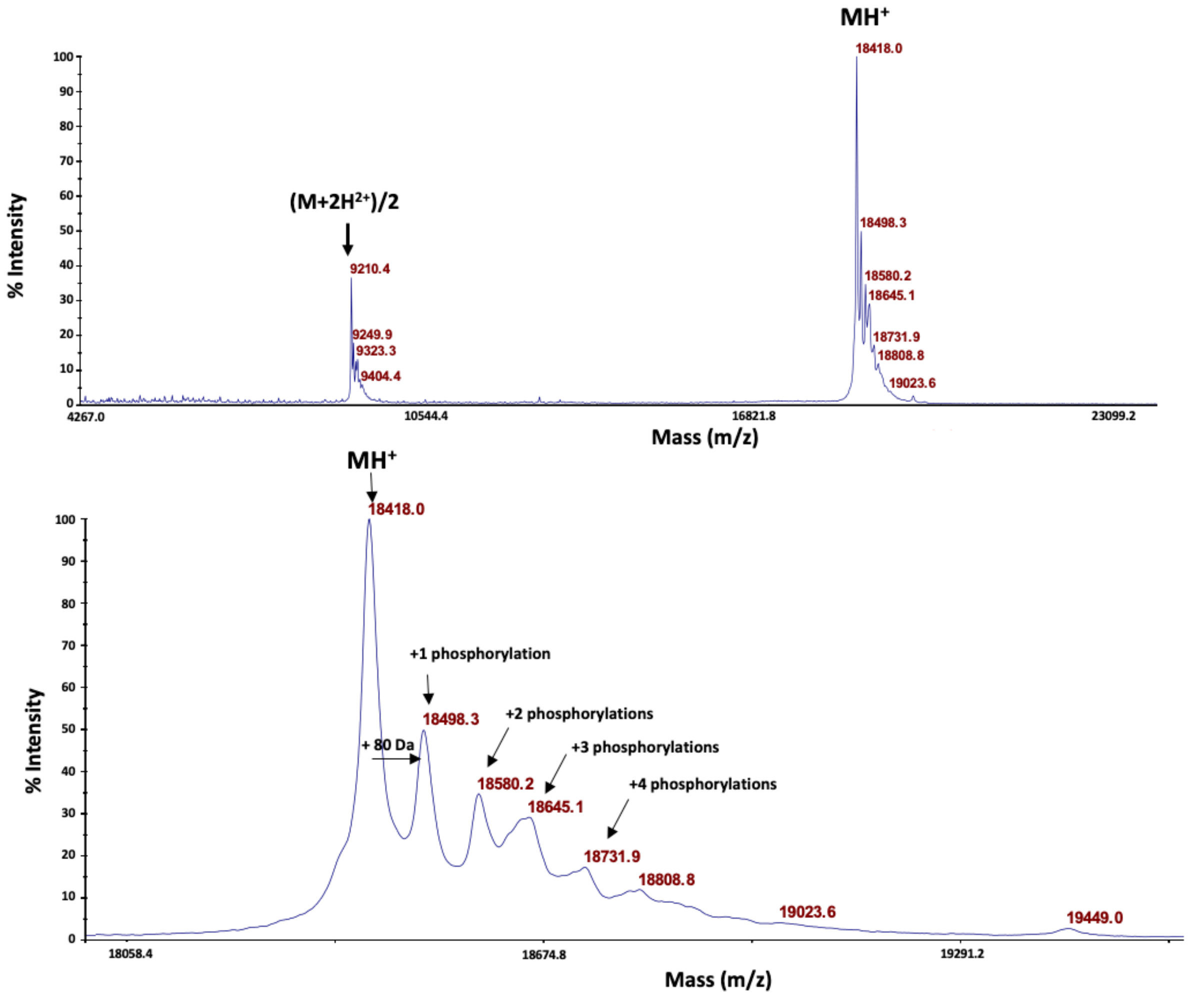

Figure S13. MALDI-TOF Mass spectrometry analysis of ^{Ecoli}PreS$_{tag}$ co-expressed with MAPK14. The full spectrum (top) and the zoom (bottom) correspond to the analysis of the purified fractions as shown in Figure S12. The first peak from the left corresponds to the mass of the protein (18418.0 Da). The additional peaks at around +80 Da correspond to cumulative phosphorylation events.

Ser6 + Thr7 or Ser8

Peptide [1-24] : MH+ : 2718.1796 Th - **MSMS on** m/z 906.73138 (3+ ; 1.72 ppm)

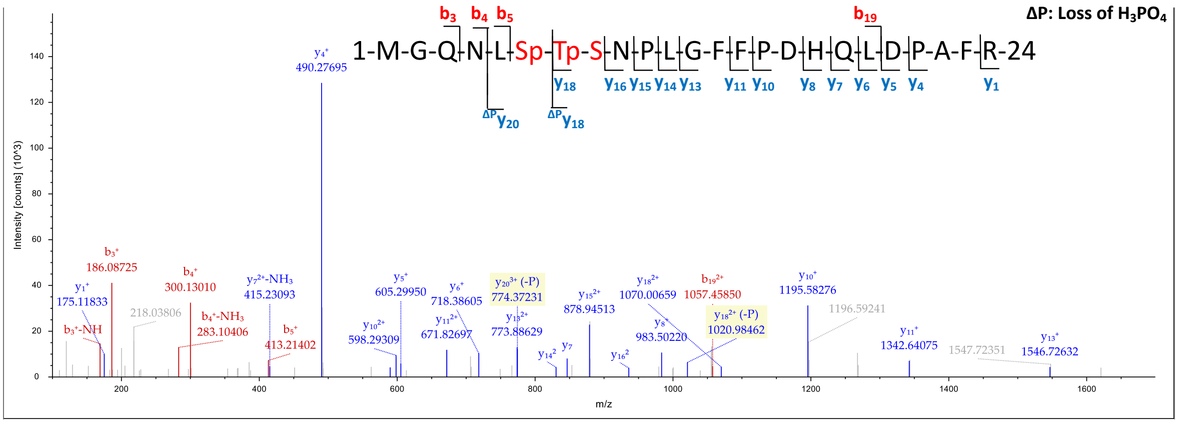

Thr27

Peptide [25-38] : MH+ : 1683.68144 Th - **MSMS on** m/z 842.34436 (2+ ; 0.92 ppm)

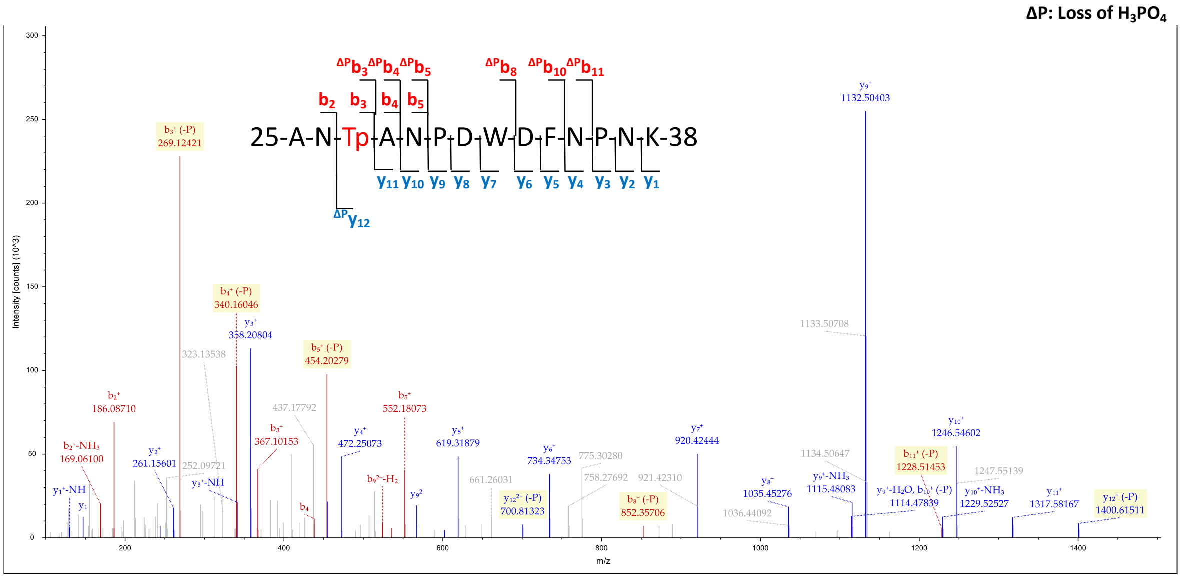

Ser90 or Thr95

Peptide [89-102] : MH⁺ : 1613.82126Th - **MSMS on** m/z 538.61194 (3+ ; 3.3 ppm)

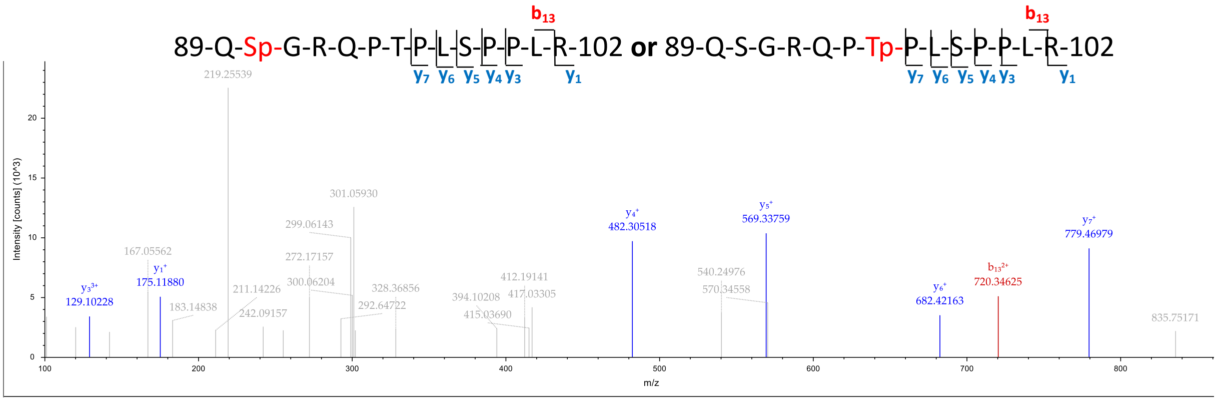

Ser98

Peptide [93-101] : MH⁺ : 1185.60271Th - **MSMS on** m/z 593. 30499 (2+ ; -0.04 ppm)

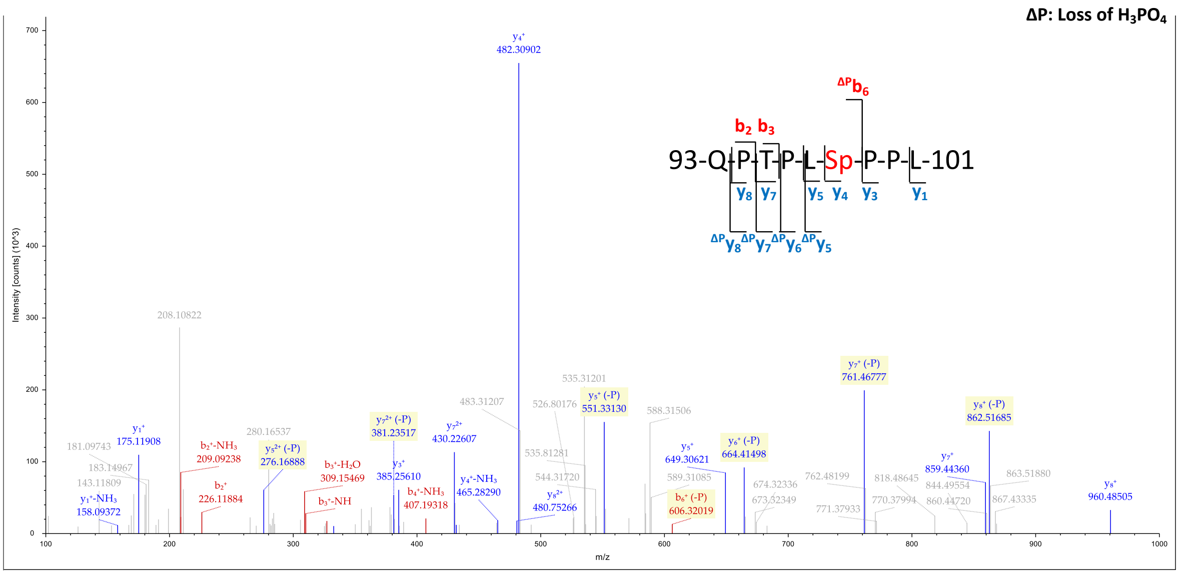

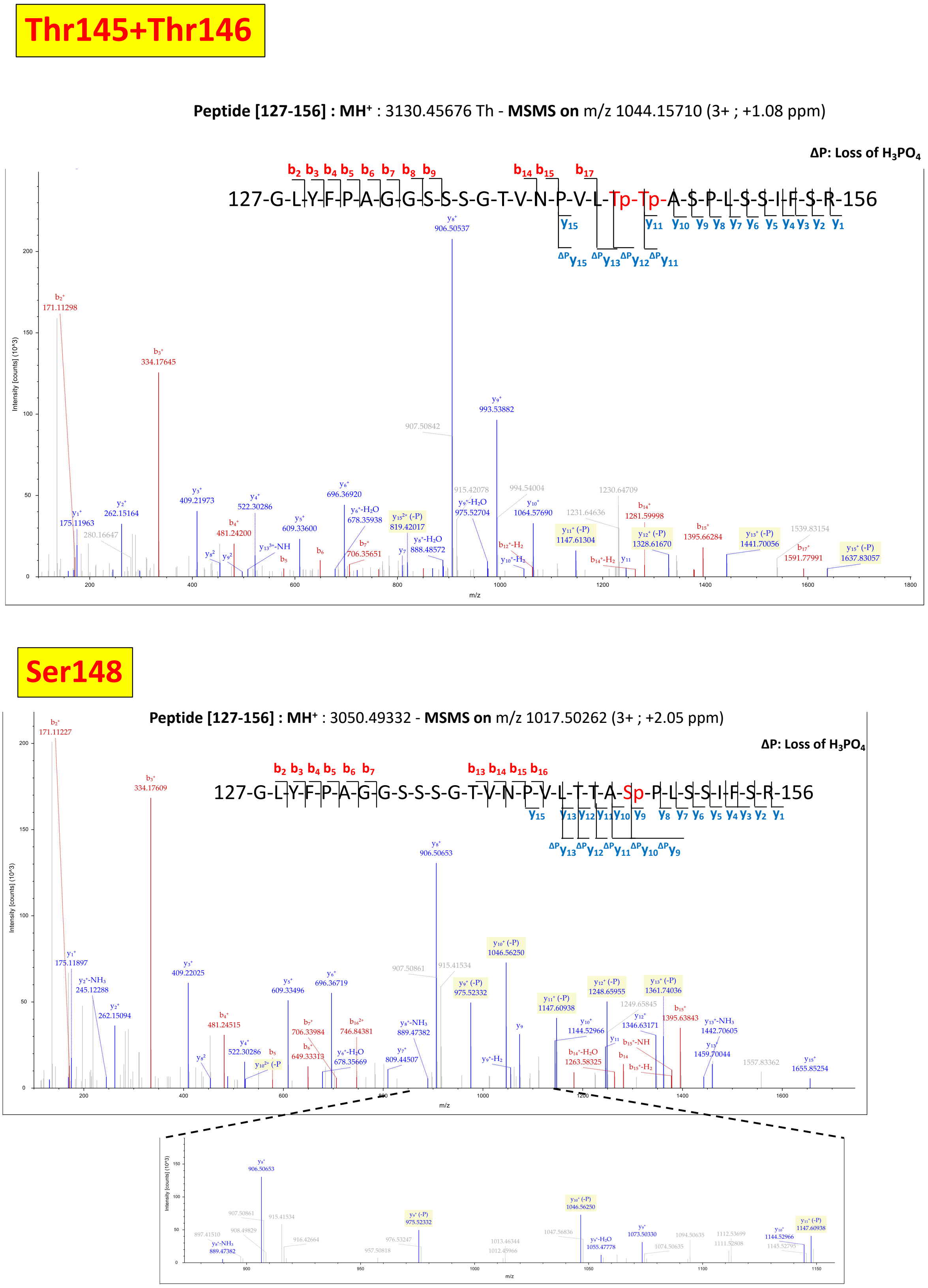

Figure S14. LC-MS/MS Mass spectrometry analysis identifying phosphorylation sites of $^{E.coli}$PreS$_{tag}$ co-expressed with MAPK14. Shown spectra correspond to the analysis of the band from the protein purification as shown in Figure S12. The confirmed phosphorylation sites are indicated in red for residues S6, T27, T95 (or S90), S98, T145, T146 and S148.

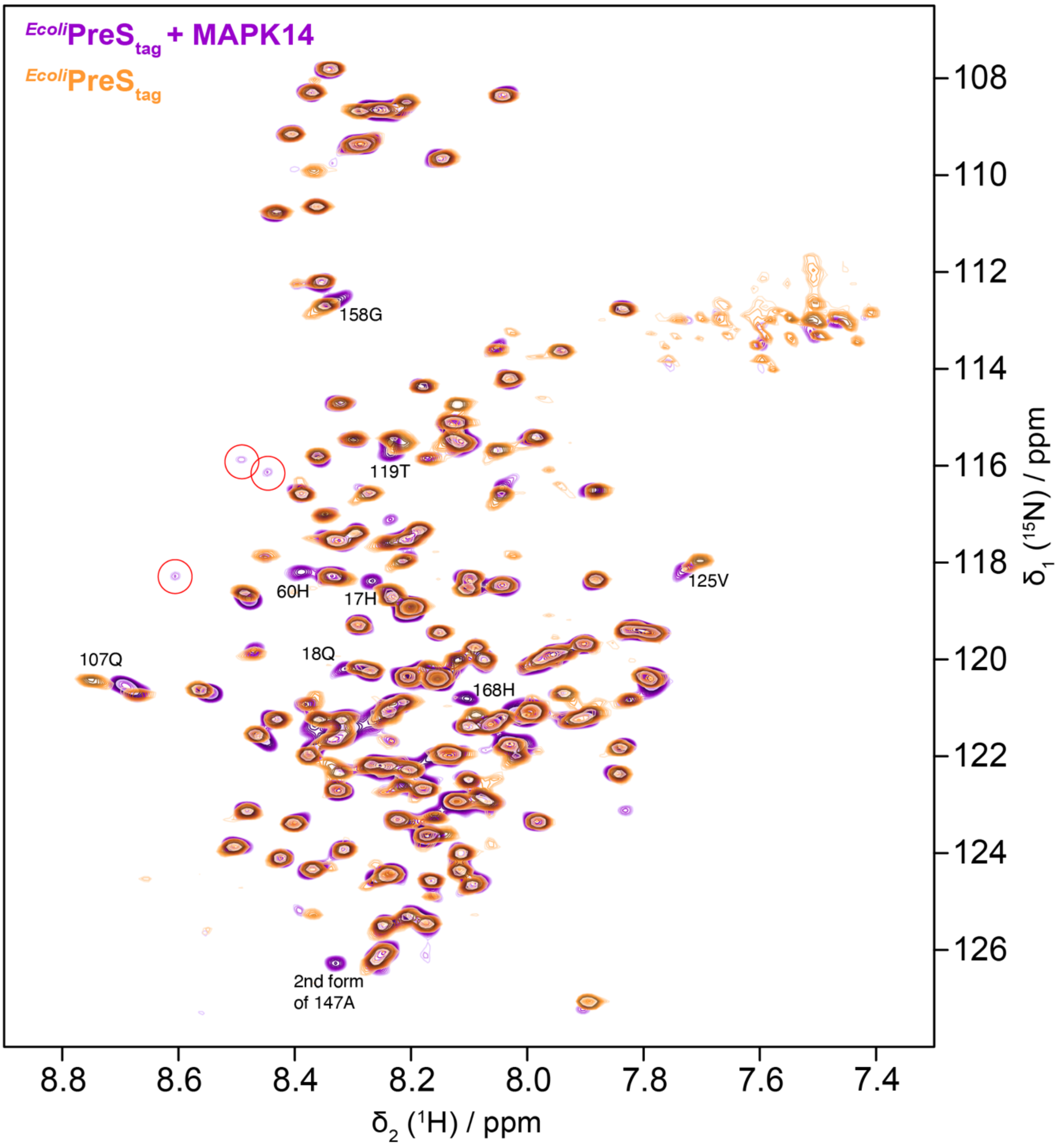

Figure S15: Comparison of HN-Best-TROSY spectra of $^{E.coli}$PreS$_{tag}$ with (purple) and without (orange) MAPK14 co-expression do not reveal phosphorylated residues. Some small chemical shift differences are observed between the two samples, for which peak assignment is labeled in black. However, no additional peaks corresponding to phosphorylated residues are observed. The 3 peaks surrounded by red circles show too low intensity to reveal any Cβ resonances in the 3D spectra.

```
        10        20        30        40        50        60        70        80
....:....|....:....|....:....|....:....|....:....|....:....|....:....|....:....|
                               6                   27
--------mg-gw--sskprkgMGtN----LSvpNPLGFFPDHQLDPAFgANsnNPDWDfNPnKDhWPdANkVGvG-AFG
        --m--  -----q-w q     h ts     l g     l k  tes    l kh  n teshq  a  sy
           al  lwtvp-e  k        t               r   a     h ti  t  a        g
            p   p t l                                r        k  q  q
                    r                                s           p  m
                                                     d           s

        90       100       110       120       130       140       150       160
....:....|....:....|....:....|....:....|....:....|....:....|....:....|....:....|
  57            67                           95 98
pGFTPPHGG----lLGWSPQAQGiLtTvpaaPPPASTNRQSG-RQPTPiSPPLRd--tHPQAMQWNSTtFHQaLq-DPRV
l li    s    i         liqslstn        rl   k   lt    n  s    ik   sa   t l n k
        n    v         m a   vi         t        v            v     q
                       t k    d         v
                       v      v
                              t

       170       180       190       200       210       220       230       240
....:....|....:....|....:....|....:....|....:....|....:....|....:....|....:....|
                    148
RgLYfPAGGSSSGTVnPvpttaSpiSSIfsrtGDPapNMEnitSGfLGPLLVLQAGFFLLTrILTIPQSLDSWWTSLNFL
ka  l       eils aqniv atl tlaki glvl  dsta  l     a   vcss  k     k         s
             aq        hl   slt     t     s
              a        l            m
                       s

       250       260       270       280       290       300       310       320
....:....|....:....|....:....|....:....|....:....|....:....|....:....|....:....|

GGtpvClGQNSQSptSnHSPTsCPPiCPGYRWMCLRRFIIFLfILL-LCLIFLLVLLDYQGMLPVCPLiP--G----s--
 eatt p   l  qi s ll c   t       y        c             v c         l        t
  s g r
  p a
  l k
  v

       330       340       350       360       370       380       390       400
....:....|....:....|....:....|....:....|....:....|....:....|....:....|....:....|
                                                        .
-s----TTST--GPCkTCTtpAQGtSMfPSCCCTKPsDGNCTCIPIPSSWAFakfLWEWASvRFSWLSLLVPFVQWFVGL
 t       v     r  mitv  n  y     s  t              lgry      a        aq    ca
         a         sl                                v
                   a

       410       420       430       440
....:....|....:....|....:....|....:....|.

SPTVWLSvIWMMWyWGPsLYnIlsPFlPLLPIFFCLWVYI-
  i   laml i f  rn cstvn  i      cy  as
                 r     r  m      l
                 x
```

Figure S16: Residue repertoire of L as derived from the hepatitis B data base (HBVDB, https://hbvdb.lyon.inserm.fr/HBVdb/HBVdbDataset?view=/data/proteins/repertoires/all_LHBs.rep&seqtype=2).

constructs	preS1-tag		preS-tag		tag-preS		tag-L
	unlabeled	^{15}N labeled	unlabeled	^{15}N^{13}C^{2}H labeled	unlabeled	^{15}N^{13}C^{2}H labeled	unlabeled
number of wells	1	2 x 6	1	2 x 6	1	2 x 6	1
colums size	1 mL	2 x 1 mL	0.2 mL	2 x 1 mL	0.2 mL	2 x 1 mL	0.2 mL
detergent expression	none	none	none	MNG-3	none	MNG-3	Brij-58
detergent purification	none	DDM	DDM	DDM	none	DDM	DDM
protein quantities	0.43 mg	1 mg	0.11 mg	0.69 mg	0.42 mg	0.86 mg	0.06 mg
yield per mL WGE used	0.28 mg/mL	0.34 mg/mL	0.46 mg/mL	0.46 mg/mL	1.7 mg/mL	0.57 mg/mL	0.25 mg/mL

Table S1: WG-CFPS sample preparation. Number of wells refers to the number of six-well plate wells used for the reaction; detergent expression denotes the detergent added during the reaction; detergent purification denotes the detergent used upon purification; yield corresponds to the produced protein quantity from 1 ml wheat-germ extract.

	$PreS1_{tag}$	$PreS_{tag}$	$_{tag}PreS$	$^{Ecoli}PreS_{tag}$	$^{Ecoli}PreS_{tag}$+MAPK
Number of residues (tag)	124 (16)	177 (14)	178 (16)	172 (10)	172 (10)
Theoretical pI	6.38	6.97	6.97	8.46	8.46
Labeling	^{2}H-^{13}C-^{15}N	^{2}H-^{13}C-^{15}N	^{2}H-^{13}C-^{15}N	^{13}C-^{15}N	^{13}C-^{15}N
C (μM)	150	120	60	50	50
Buffer	20 mM HEPES	20 mM PO_4	50 mM PO_4	20 mM PO_4	20 mM PO_4
pH	7.5	6	6	6	6
Salt	50 mM NaCl	50 mM NaCl	50 mM NaCl	50 mM NaCl	50 mM NaCl
	**Experimental time of 2D and 3D spectra**				
Magnetic field (MHz)	950	950	600	600	600
NMR tube	3 mm	3 mm	5 mm shigemi	3 mm	5 mm
2D Best-Trosy	47 min	1h15	40 min	2h17	32 min
3D HNCA	/	/	7h43	/	15h21
3D HNCACB	2 days 14h	3 days	18h	1 day 16h30	11h02
3D HNcoCACB	1 day 15h30	1 day 10h15	1 day 21h30	1 day 12h	
3D HNCO	7h20	6h43	7h12	14h	
3D HNcaCO	22h	8h36	7h	6h	
	**Assignment completeness:**				
HN	80/104	141/152	131/153	140/147	140/147
NH (including Pro)	80/124	141/177	131/178	140/172	140/172
CO	100/124	165/177	154/178	157/172	
CA	97/124	166/177	156/178	161/172	
CB	84/113	149/161	132/162	146/156	
Backbone assignment (H_N, N_H, CO, Ca) (%)	**75 %**	**90 %**	**83 %**	**90 %**	

Table S2: NMR sample details and assignment completeness for the 4 PreS NMR samples. For $^{Ecoli}PreS_{tag}$ with MAPK14, short 3D spectra were acquired to confirm the HN assignment since it is highly similar to the sample produced without MAPK14.

Random coil shifts	HN	N	C′	Cα	Cβ
Ser	8.59	117.63	174.80	58.60	63.71
pSer	9.13	118.87	174.77	58.24	65.73

Residue	Construction	HN	15N	CO	Ca	Cb
Ser6	PreS1$_{tag}$	8.65	119.80	171.87	55.70	64.12
	PreS$_{tag}$	8.38	116.17	175.04	58.16	63.16
	PreS$_{tag}$'	8.41	116.46	175.02	58.14	63.14
	$_{tag}$PreS	8.36	116.06	175.03	58.14	63.17
Ser8	PreS$_{tag}$	8.24	117.34	176.75	57.96	63.25
	$_{tag}$PreS	8.22	117.32	174.05	57.9	63.21
Ser67	PreS1$_{tag}$	7.85	119.05	172.20	55.44	63.20
	PreS$_{tag}$	7.83	119.29		55.38	63.12
	$_{tag}$PreS	7.84	118.99	172.23	55.43	63.06
Ser85	PreS$_{tag}$	8.36	114.71	175.09	58.09	63.26
	PreS$_{tag}$'	8.37	115.05	174.79	57.78	63.32
	$_{tag}$PreS	8.37	114.82	175.04	57.93	63.20
	$_{tag}$PreS’	8.37	115.08	174.79	57.78	63.23
Ser90	PreS$_{tag}$	8.39	116.97	175.13	58.30	63.28
	PreS$_{tag}$'	8.64	118.34	174.99	58.21	63.32
	$_{tag}$PreS	8.38	116.98	175.12	58.23	63.24
Ser98	PreS1$_{tag}$	8.24	117.71	171.96	56.03	62.70
	PreS$_{tag}$	8.27	118.01	172.06	56.03	62.61
	PreS$_{tag}$'	8.51	118.74	179.18	55.22	64.65
	$_{tag}$PreS	8.27	118.01	174.37	55.92	62.78
	$_{tag}$PreS’	8.50	118.73		55.20	64.59
Ser113	PreS$_{tag}$	8.14	115.74	174.73	58.14	63.17
	$_{tag}$PreS	8.14	115.76	174.74	58.04	63.13
Ser135	PreS$_{tag}$	8.34	115.45	174.96	57.96	63.37
	$_{tag}$PreS	8.34	115.47	174.95	57.90	63.28
Ser136	PreS$_{tag}$	8.48	117.76	174.84	52.93	63.21
	$_{tag}$PreS	8.48	117.80	174.82	58.06	63.18
Ser137	PreS$_{tag}$	8.38	117.45	175.08	58.29	63.21
	$_{tag}$PreS	8.37	117.46	175.03	58.29	63.20
Ser148	PreS$_{tag}$	8.34	116.63	173.23	56.03	62.85
	$_{tag}$PreS	8.34	116.67	175.99	55.93	62.83
Ser151	PreS$_{tag}$	8.18	115.62	174.52	58.10	63.28
	$_{tag}$PreS	8.17	115.57	174.50	58.11	63.14
Ser152	PreS$_{tag}$	8.24	117.33	174.07	57.91	63.30
	$_{tag}$PreS	8.23	117.32	174.04	57.95	63.21

Table S3: NMR chemical shifts of serine residues. Table of chemical shifts (in ppm) of unphosphorylated and phosphorylated serine residue in random-coil conformation taken from (Hendus-Altenburger et al., 2019), compared to chemical shifts of serine residues in PreS1 and PreS NMR samples. Chemical shifts indicating possible phosphorylation sites are written in red. The ‘ corresponds to the 2nd resonances observed in some region of PreS samples.

random coil shifts	HN	N	C′	Cα	Cβ
Thr	8.41	116.38	174.65	62.15	69.88
pThr	9.09	119.10	174.57	62.99	72.45

Residue	Construction	HN	15N	CO	Ca	Cb
Thr7	PreS$_{tag}$	8.16	115.07	174.63	61.45	69.13
	$_{tag}$PreS	8.13	114.90	174.60	61.44	69.13
Thr27	PreS1$_{tag}$	7.99	113.61	174.11	61.54	69.27
	PreS$_{tag}$	7.99	113.67	174.19	61.49	69.23
	$_{tag}$PreS	7.99	113.68	174.17	61.43	69.26
Thr40	PreS1$_{tag}$	7.88	112.73	174.04	61.43	69.51
	PreS$_{tag}$	7.88	112.84	174.11	61.51	69.45
	$_{tag}$PreS	7.87	112.87	174.09	61.41	69.46
Thr57	PreS1$_{tag}$	8.13	119.78	171.72	58.93	69.57
	PreS$_{tag}$	8.14	119.60	171.78	58.89	69.51
	$_{tag}$PreS	8.14	119.58	171.81	58.96	69.57
	^{Ecoli}PreS$_{tag}$	8.07	120.01	171.70	58.87	69.63
Thr76	PreS1$_{tag}$	8.14	115.34	174.22	61.48	69.43
	PreS$_{tag}$	8.14	115.63		61.42	69.41
	$_{tag}$PreS	8.14	115.35	174.25	61.44	
Thr86	PreS$_{tag}$	8.19	115.32	174.46	61.56	69.26
	$_{tag}$PreS	8.18	115.22	174.46	61.46	69.24
Thr95	PreS1$_{tag}$	8.34	117.21	173.04	59.58	69.37
	PreS$_{tag}$	8.34	117.39	173.10	59.57	69.29
	$_{tag}$PreS	8.34	117.38	173.06	59.50	69.37
Thr104	PreS1$_{tag}$	8.07	113.23	173.33	61.34	69.61
	PreS$_{tag}$	8.11	113.70	174.21	61.38	69.19
	$_{tag}$PreS	8.11	113.72	174.21	61.37	69.26
Thr114	PreS$_{tag}$	8.21	115.48	174.54	61.59	69.30
	PreS$_{tag}$'			174.96	62.14	69.14
	$_{tag}$PreS	8.19	115.46	174.51	61.49	69.25
	$_{tag}$PreS′			174.96	62.06	69.11
Thr115	PreS$_{tag}$	8.06	116.32	173.27	61.32	69.44
	PreS$_{tag}$'	8.02	115.53	174.33	61.90	69.17
	$_{tag}$PreS	8.06	116.33	173.23	61.27	69.42
	$_{tag}$PreS′	8.02	115.56	174.29	61.76	69.19
Thr119	PreS$_{tag}$	8.53	117.55	174.46	61.76	69.37
	$_{tag}$PreS	8.53	117.58	174.45	61.65	69.38
Thr139	PreS$_{tag}$	8.08	114.34	174.23	61.53	69.46
	$_{tag}$PreS	8.07	114.36	174.46	61.45	69.45
Thr145	PreS$_{tag}$	8.18	115.07	174.66	61.43	69.33
	$_{tag}$PreS	8.17	115.04	174.65	61.26	69.35
Thr146	PreS$_{tag}$	8.11	115.93	174.12	61.31	69.39
	$_{tag}$PreS	8.11	115.97	174.09	61.29	69.38

Table S4: NMR chemical shifts of threonine residues. Table of chemical shifts (in ppm) of unphosphorylated and phosphorylated threonine residues in random-coil conformation taken from (Hendus-Altenburger et al., 2019), compared to chemical shifts of threonine residues in PreS1 and PreS NMR samples. Typical chemical shifts indicating possible phosphorylation sites are written in red. The ' corresponds to the 2nd resonances observed in some region of PreS samples. For T57, the ^{Ecoli}PreS (without MAPK14) chemical shifts were added to highlight the fact that the unusual ^{15}N chemical shift is not due to phosphorylation.

Supplementary References

Hendus-Altenburger, R., Fernandes, C. B., Bugge, K., Kunze, M. B. A., Boomsma, W., and Kragelund, B. B. (2019). Random coil chemical shifts for serine, threonine and tyrosine phosphorylation over a broad pH range. *J Biomol Nmr* 73, 713–725. doi:10.1007/s10858-019-00283-z.

Wang, Y., and Jardetzky, O. (2002). Probability-based protein secondary structure identification using combined NMR chemical-shift data. *Protein Sci* 11, 852–861. doi:10.1110/ps.3180102.
